# Perinatal Redox Dysregulation in Gestational Tobacco Exposure: Compartment-Specific Composite Indices and Their Association with Birth Outcomes—A Prospective Cohort Study

**DOI:** 10.3390/antiox15080996

**Published:** 2026-08-11

**Authors:** Zsuzsánna Simon-Szabó, Sándor Pál, Enikő Nemes-Nagy, Lóránd Dénes, Erzsébet Májai, Margit Solymár, Zsuzsanna Faust, Erika-Gyöngyi Bán, Ana Maria Fárr, Florina Gliga, Hajnal Finta, Manuela Cucerea

**Affiliations:** 1 Pathophysiology Department, George Emil Palade University of Medicine, Pharmacy, Science, and Technology of Târgu Mureș, 540142 Târgu Mureș, Romania; zsuzsanna.simon-szabo@umfst.ro (Z.S.-S.);; 2 Department of Laboratory Medicine, Department of Transfusion Medicine, Medical School, University of Pécs, 7622 Pécs, Hungary; 3 Chemistry and Medical Biochemistry Department, George Emil Palade University of Medicine, Pharmacy, Science, and Technology of Târgu Mureș, 540142 Târgu Mureș, Romania; eniko.nemes-nagy@umfst.ro; 4 Anatomy and Embriology Department, George Emil Palade University of Medicine, Pharmacy, Science, and Technology of Târgu Mureș, 540142 Târgu Mureș, Romania; 5 Toxicology and Biopharmacy Department, George Emil Palade University of Medicine, Pharmacy, Science, and Technology of Târgu Mureș, 540142 Târgu Mureș, Romania; erzsebet.fogarasi@umfst.ro; 6 Pharmacology Department, George Emil Palade University of Medicine, Pharmacy, Science, and Technology of Târgu Mureș, 540142 Târgu Mureș, Romania; 7 Department of Public Health and Health Management, George Emil Palade University of Medicine, Pharmacy, Science, and Technology of Târgu Mureș, 540142 Târgu Mureș, Romania; 8 Department of Medical Evaluation and Statistics, Emergency County Hospital of Târgu Mureș, 540142 Târgu Mureș, Romania; 9 Neonatology Department, George Emil Palade University of Medicine, Pharmacy, Science, and Technology of Târgu Mureș, 540142 Târgu Mureș, Romania; manuela.cucerea@umfst.ro

**Keywords:** composite indices, gestational tobacco exposure, global redox index, low birth weight, nitrosative stress index, oxidative stress index, pregnancy, preterm birth

## Abstract

Background: Gestational tobacco exposure is a major modifiable risk factor for adverse pregnancy outcomes; how smoking-induced redox perturbations transmit across the placental barrier to the fetal compartment, and mediate neonatal outcomes, remains incompletely understood. Methods: In a prospective cohort of 66 maternal-neonatal dyads (24 smokers, 42 non-smokers) from Târgu-Mures, six redox biomarkers (nitrite, nitrate, malondialdehyde, reduced, oxidized glutathione (GSH, GSSG) and GSH/GSSG ratio) were quantified in paired maternal and cord blood and converted to SI units, and pathway-specific composite indices (oxidative and nitrosative stress index–OSI, NSI, global redox index–GRI) were derived via standardized Z-scores of natural-log-transformed concentrations. Associations with preterm birth (PTB) and low birth weight (LBW) were assessed via multivariable regression. Benjamini–Hochberg false discovery rate (FDR) correction was applied within predefined test families. Results: Tobacco exposure was associated with nominally significant depletion of the Neonatal NSI (mean difference = −0.842; 95% CI: −1.567 to −0.117; *p* = 0.024; FDR-adjusted *p* = 0.142), while maternal indices showed no significant group differences. Lower Maternal GRI and Neonatal NSI were independently associated with lower birth weight and higher odds of preterm birth (aOR = 0.35 and 0.52, respectively; both *p* < 0.05). Maternal and neonatal nitrite concentrations were positively correlated (FDR-adjusted *p* = 0.002) and oxidized glutathione showed an inverse maternal-neonatal correlation (FDR-adjusted *p* = 0.001), whereas the maternal and neonatal NSI was not significant after FDR-correction (FDR-adjusted *p* = 0.078) Conclusions: Gestational tobacco exposure was associated with depletion of the neonatal nitrosative stress index. Independently, lower maternal and neonatal composite redox indices were associated with lower birth weight and higher odds of preterm birth. Composite indices may offer an integrated assessment of perinatal redox status, but without external validation warrant confirmation in larger cohorts.

## 1. Introduction

Gestational tobacco exposure remains one of the most consequential preventable risk factors for adverse maternal and neonatal outcomes worldwide. Literature data shows that approximately 30–49% of Romanian women smoke before pregnancy. Concerningly, about half of them continued to smoke after learning about their pregnancy, which, according to the results of some local studies, indicates that approximately 15% to nearly 40% of pregnant participants smoke [1]. Epidemiological evidence has consistently demonstrated associations between active smoking during pregnancy and a spectrum of adverse outcomes, including fetal growth restriction, reduced mean birth weight, low birth weight, preterm birth, placental abruption, stillbirth, and neonatal death [1,2,3,4]. A systematic review and meta-analysis of 142 cohort and case–control studies reported that individuals who smoked during pregnancy had an increased risk of perinatal death (summary Relative Risk–RR 1.33; 95% Confidence Interval–CI 1.25–1.41), stillbirth (summary RR 1.46; 95% CI 1.38–1.54), and neonatal death (summary RR 1.22; 95% CI 1.14–1.30) compared with non-smokers [2]. A dose–response analysis of over 25 million mother–infant pairs further demonstrated that even very low-intensity smoking (1–2 cigarettes per day) during either the first or second trimester was associated with a significantly increased risk of preterm delivery, suggesting that no safe threshold of gestational tobacco exposure exists [4]. Exposure to maternal and secondhand tobacco smoke has been associated with preeclampsia, while pregestational smoking increases the risk of gestational diabetes (GDM), meanwhile smoking-related insulin resistance and dyslipidemia contribute to adverse maternal metabolic profiles—although meta-analytic evidence regarding the link between active smoking during pregnancy and GDM remains inconsistent [5,6,7,8,9]. The pathophysiological mechanisms underlying these associations are thought to be complex and multifactorial. Tobacco smoke contains over 7000 chemical compounds, many of which are potent generators of reactive oxygen species (ROS) and reactive nitrogen species (RNS) through activation of cytochrome P450 pathways and direct radical delivery [10,11]. Nicotine and carbon monoxide, two of the most pharmacologically active constituents, have been implicated in placental vascular dysfunction through distinct but potentially synergistic mechanisms. Nicotine readily crosses the placental barrier, with fetal concentrations typically exceeding maternal levels by approximately 15% and has been shown to inhibit trophoblast migration and impair spiral artery remodeling [12,13]. Carbon monoxide forms carboxyhemoglobin, which may reduce oxygen delivery to fetal tissues, and has been associated with suppression of Wingless-related integration site (WNT) signaling and trophoblast proliferation in placental tissue [12,14]. Together, these constituents may contribute to a state of chronic oxidative and nitrosative imbalance at the maternal–fetal interface. Tobacco smoke sustains a low-grade systemic inflammatory state, amplifying reactive oxygen and nitrogen species, and delivers direct radicals into the bloodstream. Pregnancy is already associated with an increased inflammatory status, which may compound the redox burden at the maternal-fetal interface [11,15,16].

Pregnancy itself is a physiological state characterized by heightened susceptibility to oxidative stress, driven by increased metabolic demand and a systemic inflammatory response that elevates circulating ROS and RNS [11,17]. Under normal conditions, endogenous antioxidant defense systems, like glutathione (GSH), superoxide dismutase (SOD), catalase (CAT), and glutathione peroxidase (GPx), maintain redox homeostasis [11]. However, when the generation of reactive species exceeds antioxidant capacity, oxidative stress ensues, potentially contributing to placental dysfunction, impaired trophoblast invasion, defective vasculogenesis, and compromised nutrient transfer to the fetus [18,19]. In pregnancies complicated by tobacco exposure, this physiological oxidative burden may be further amplified by exogenous pro-oxidants delivered through cigarette smoke [10,20,21].

A growing amount of evidence has linked individual biomarkers of oxidative and nitrosative stress to adverse perinatal outcomes. Chełchowska et al. demonstrated that maternal smoking during pregnancy was associated with elevated plasma malondialdehyde (MDA) and depleted total radical-trapping antioxidant parameters (TRAP) in both maternal and cord blood [20]. The same research group subsequently reported that the GSH/GSSG ratio—widely regarded as an index of cellular redox status—was significantly lower in both smoking mothers and their neonates compared with non-smoking pairs, accompanied by decreased glutathione reductase (GR) levels [21]. In the nitrosative domain, maternal smoking has been associated with reduced serum nitric oxide (NO), decreased endothelial NO synthase (eNOS) concentrations, and elevated inducible NOS (iNOS) and total oxidant capacity (TOC) in maternal blood [22]. At the fetal level, Andersen et al. observed that eNOS activity and eNOS concentration in the umbilical veins of smokers were significantly lower than in nonsmokers; these changes could be avoided by smoking cessation as early as the first trimester of pregnancy [23]. Zahorán et al. further characterized endothelial-mediated oxidative stress and NOS3 deactivation in umbilical cord vessels of neonates born to heavy-smoking mothers, with vessel-specific alterations proportional to the level of exposure [15]. Nitric oxide is a central regulator of uteroplacental and umbilical vascular tone, promoting vasodilation and adequate fetal perfusion; disturbance of its synthesis or bioavailability can impair spiral-artery remodeling and fetal growth, providing the physiological rationale for examining nitrosative markers alongside oxidative ones [24,25,26].

Despite these advances, standard approaches in perinatal redox research have typically relied on individual biomarkers assessed independently of one another. However, individual markers often exhibit high inter-individual variability and may not adequately capture the coordinated, pathway-specific stress responses that operate within distinct biological compartments [27,28]. The American Heart Association (AHA) Scientific Statement on Measurement of Reactive Oxygen Species has emphasized that no single biomarker has been identified as an ideal index of oxidative status, and has recommended that measurements of multiple independent indices of oxidative stress be employed in human studies [27]. Furthermore, the AHA recommends that values of antioxidants and oxidation products be reported in absolute molar concentrations to facilitate inter-laboratory and inter-tissue comparisons [27].

To address these limitations, the composite indices integrating both pro-oxidant and antioxidant markers have been proposed. The OXY-SCORE, developed by Veglia et al., combines plasma MDA, GSSG/GSH ratio, urinary isoprostanes, GSH, tocopherols, and individual antioxidant capacity into a single standardized score, and has been shown to discriminate between cardiovascular disease patients and healthy controls, as well as between age and gender groups [29,30]. Sánchez-Rodríguez and Mendoza-Núñez, in a comprehensive review of oxidative stress indices, concluded that the Oxidative Stress Index, OXY-SCORE, and Oxidative Stress Score (OSS) have demonstrated reliability, practicality, and clinical utility across diverse clinical conditions [31]. However, these composite approaches have been predominantly applied in cardiovascular and aging research, and their application to perinatal medicine, particularly in the context of gestational tobacco exposure, was never adopted.

The present study sought to extend this composite index approach to the perinatal setting by constructing pathway-specific indices: an Oxidative Stress Index, a Nitrosative Stress Index, and a combined Global Redox Index, from standardized Z-scores of six primary redox biomarkers (nitrite, nitrate, MDA, GSH, GSSG, and GSH/GSSG ratio) measured simultaneously in maternal and cord blood. This dual-compartment design was motivated by evidence that the placenta functions as a selective barrier that may differentially modulate the transfer of various redox-active species between maternal and fetal circulations, and that maternal and fetal oxidative stress profiles may not necessarily mirror one another [20,21,32,33].

The primary objective was to evaluate whether gestational tobacco exposure is associated with alterations in these composite stress indices in maternal and neonatal compartments separately. Specifically, the study tested: (1) the hypothesis that active gestational smoking is associated with elevated maternal and neonatal composite oxidative stress indices (MOSI and NOSI) (Primary Hypothesis), and (2) secondary hypothesis was to determine whether these composite stress indices are associated with secondary clinical endpoints (PTB, LBW, and birth weight) in multivariable regression models adjusted for clinical covariates.

## 2. Materials and Methods

### 2.1. Study Design and Participant Selection

This prospective cohort study conducted between November 2019–October 2020, enrolled consecutive mother–newborn dyads at the tertiary-level Obstetrics-Gynecology Clinic I of the Emergency County Clinic Hospital (ECCH) in Târgu Mureș, Romania. The study was conducted in accordance with the Declaration of Helsinki, and the protocol was approved by the Institutional Ethics Committee of ECCH Târgu Mureș and the Ethics Committee of G.E.P. UMPhST Târgu Mureș. The laboratory analyses were funded by G.E.P. UMPhST through grant No. 171/6/09.01.2024. The frozen plasma samples were analyzed in 2024. Written informed consent was obtained from all participating mothers prior to sample collection.

The study population comprised pregnant women classified into two groups based on their self-reported smoking status during pregnancy. Paired biological samples (maternal peripheral venous blood and umbilical cord blood) were collected at the time of delivery. Exclusion criteria included multiple gestations, pre-existing maternal autoimmune diseases, severe maternal chronic diseases (e.g., gestational diabetes mellitus, chronic renal failure), and cases of perinatal asphyxia (defined as a 5-min Apgar score < 5). In addition, cases of congenital cardiac malformations were excluded to focus strictly on gestational tobacco exposure. Detailed data on tobacco exposure (such as pack-years, daily smoking, and product types) were not collected. The absence of detailed data is attributable to the self-reporting nature of the study, which introduces the possibility of individual subjectivity and may lead to an under-reporting or misinterpretation of biological data. The primary analytical cohort comprised 66 dyads (24 smokers, 42 non-smokers).

### 2.2. Biospecimen Collection and Processing

Maternal peripheral venous blood samples were obtained via venipuncture prior to delivery. Neonatal cord blood samples were obtained from the umbilical vein immediately following umbilical cord clamping. All blood samples were collected in ethylenediaminetetraacetic acid (EDTA) tubes. Samples were centrifuged at 3000 rpm for 10 min at 4 °C within 30 min of collection to separate plasma. Plasma aliquots were immediately frozen and stored at −80 °C until laboratory analysis.

### 2.3. Laboratory Analysis of Biomarkers

All laboratory analyses were performed by researchers blinded to the participants’ clinical characteristics and exposure status.

Plasma malondialdehyde (MDA) was quantified as its thiobarbituric acid derivative by HPLC with visible detection [34]. Thawed plasma (200 µL) was mixed with 600 µL acetonitrile (VWR International SAS, Fontenay-sous-Bois, France) and centrifuged at 5000 rpm for 5 min. An aliquot of the supernatant (300 µL) was combined with 450 µL thiobarbituric acid solution (Sigma-Aldrich, St. Gallen, Switzerland) (4 mg/mL) and 750 µL sulfuric acid (H_2_SO_4_ 50 mM) (Chemical Company, Iași, Romania). The mixture was vortexed, heated at 100 °C for 60 min, cooled on ice, and transferred to an HPLC vial.

Chromatographic separation was performed using a Merck-Hitachi HPLC system (Merck KGaA, Darmstadt, Germany) equipped with a diode-array detector and a Supelcosil LC-18 column (3 µm, 33 × 4.6 mm; Merck KGaA, Darmstadt, Germany). Other components of the system consisted of a quaternary pump (L-7100), an autosampler (L-7200), a column thermostat (L-7360), a DAD detector (L-7455), an interface (L-7000), a solvent degasser (L-7612) and a software (D-7000 HSM-Manager) (version 4.1). Isocratic elution was performed with 20 mM phosphate buffer (pH 6.0) and acetonitrile at a flow rate of 1.0 mL/min. The injection volume was 100 µL, and detection was performed at 532 nm. The retention time of the MDA derivative was 0.58 min. MDA concentrations were determined by external calibration using standards prepared from 1,1,3,3-tetramethoxypropane (Sigma-Aldrich, St. Gallen, Switzerland) over the range of 0.52–262 ng/mL [34].

Plasma nitrate and nitrite concentrations were determined simultaneously by ion-pair reversed-phase HPLC with UV–visible detection [35].

Thawed sample supernatant (200 µL) was mixed with 200 µL tetrabutylammonium hydroxide solution (5 mM, adjusted to pH 2.5 with H_2_SO_4_) and centrifuged at 8000 rpm for 10 min. An aliquot of the resulting supernatant (150 µL) was sequentially mixed with 15 µL Griess reagent A and 15 µL Griess reagent B and refrigerated until analysis. All reagents used for this procedure were obtained from Merck KGaA (Darmstadt, Germany).

Griess reagent A was prepared by dissolving 50 mg sulfanilic acid in 1.5 mL acetic acid and 3 mL ultrapure water by ultrasonication. Griess reagent B contained 4 mg 1-naphthylamine dissolved in 4 mL acetic acid. Both reagents were stored under refrigeration and used within 3 days.

Chromatographic analysis was performed using a Merck-Hitachi HPLC system equipped with a diode-array detector (the same used for the MDA assay), a LiChroCART LiChrospher 100 RP-18 analytical column (5 µm, 250 × 4 mm), and a corresponding RP-18 guard column (Merck KGaA, Darmstadt, Germany). The mobile phase comprised tetrabutylammonium hydroxide (pH 2.5), methyl alcohol and acetonitrile. The injection volume was 100 µL. Nitrate was detected directly at 222 nm, whereas derivatized nitrite was detected at 520 nm. Quantification was based on external calibration using standards processed identically to the samples over concentration ranges of 200–40,000 ng/mL for nitrate and 10–400 ng/mL for nitrite [35].

Reduced glutathione (GSH) and oxidized glutathione (GSSG) were determined by HPLC following derivatization with 5,5′-dithiobis (2-nitrobenzoic acid), or Ellman’s reagent [36]. For GSH determination, plasma samples were pretreated with Ellman’s reagent (Sigma Aldrich, St. Gallen, Switzerland) in 1:1 ratio after blood collection. 500 µL thawed plasma + reagent mixture underwent deproteinization with 150 µL trichloroacetic acid and centrifugation at 10,000 rpm for 20 min. For total glutathione determination, samples were heated at 80 °C for 30 min before trichloroacetic acid precipitation and centrifugation under the same conditions. The resulting supernatants were transferred to HPLC vials and stored until analysis. GSSG was derived from the difference between total and reduced glutathione after conversion of GSSG to GSH by heating.

Chromatographic separation was performed using a Merck-Hitachi HPLC system equipped with a diode-array detector (the same used for the MDA assay) and a Zorbax Eclipse XDB-C8 column (5 µm, 150 × 4.6 mm; Agilent Technologies, Santa Clara, CA, USA). The mobile phase comprised 20 mM phosphate buffer (pH 2.5) (prepared from anhydrous Na_2_HPO_4_ and 85% H_3_PO_4_ purchased from Merck KGaA, Darmstadt, Germany) and acetonitrile (VWR International SAS, Fontenay-sous-Bois, France), delivered at a flow rate of 1.0 mL/min. The injection volume was 50 µL, and detection was performed at 330 nm. The mean retention time of the GSH–DTNB derivative was 9.11 min. Quantification was based on external calibration with GSH and GSSG standards over the range of 0.5–100 µg/mL [36].

The method was linear over the investigated concentration range.

### 2.4. SI Unit Conversion and Ratio Calculations

To ensure compliance with standardized medical reporting, raw laboratory values were converted to SI units (μmol/L) using the following molecular weights: Nitrite (${\mathrm{NO}}_{2}^{-}$): 1 ng/mL=0.0217366 μmol/L (MW=46.0055 g/mol), Nitrate (${\mathrm{NO}}_{3}^{-}$): 1 μg/mL=16.1278 μmol/L (MW=62.0049 g/mol), Malondialdehyde (MDA): 1 ng/mL=0.0138768 μmol/L (MW=72.063 g/mol), Reduced Glutathione (GSH): 1 μg/mL=3.25394 μmol/L (MW=307.32 g/mol), Oxidized Glutathione (GSSG): 1 μg/mL=1.63231 μmol/L (MW=612.63 g/mol), the GSH/GSSG molar ratio was computed from these converted concentrations (${\mathrm{GSH}}_{\mu \mathrm{mol}/L}/{\mathrm{GSSG}}_{\mu \mathrm{mol}/L}$).

### 2.5. Formulation of Pathway-Specific Composite Indices

To evaluate cumulative pathway-specific stress, three composite indices were calculated using standardized Z-scores (scaled across the pooled population to ensure equal weighting).

Because the individual biomarker concentrations were right-skewed, each biomarker was natural-log transformed prior to standardization; Z-scores were then computed on the log scale across the pooled study population within each compartment, and the composite indices were derived from these standardized values. The corresponding indices are denoted with an ‘M’ prefix for the maternal compartment (MOSI, MNSI, MGRI) and an ‘N’ prefix for the neonatal compartment (NOSI, NNSI, NGRI). The OSI, NSI, and GRI formulations used here are original to this study, adapting the standardized Z-score composite-index concept previously applied in cardiovascular and aging research [31] to the perinatal setting.

1. Oxidative Stress Index (OSI): constructed to reflect lipid peroxidation and glutathione depletion:
  $$\mathrm{OSI}=Z_{\mathrm{MDA}}+Z_{\mathrm{GSSG}}-Z_{\mathrm{GSH}}$$
2. Nitrosative Stress Index (NSI): constructed to reflect nitric oxide metabolites:
  $$\mathrm{NSI}=Z_{{\mathrm{NO}}_{2}^{-}}+Z_{{\mathrm{NO}}_{3}^{-}}$$
3. Global Redox Index (GRI): constructed to reflect overall redox imbalance:
  $$\mathrm{GRI}=\mathrm{OSI}+\mathrm{NSI}=Z_{\mathrm{MDA}}+Z_{\mathrm{GSSG}}-Z_{\mathrm{GSH}}+Z_{{\mathrm{NO}}_{2}^{-}}+Z_{{\mathrm{NO}}_{3}^{-}}$$

### 2.6. Clinical Outcome Definitions

Preterm birth (PTB) was defined as a delivery occurring at <37.0 weeks of gestation. Low birth weight (LBW) was defined as birth weight <2500 g.

### 2.7. Statistical Analysis

Continuous clinical and demographic variables are presented as mean ± standard deviation (SD). Categorical variables are presented as absolute counts and percentages. Distributional assumptions were assessed with the Shapiro–Wilk test for all continuous variables (Supplementary Table S3). Normality was rejected for 9 of 10 raw biomarker concentrations and for 4 of the 6 composite indices; parametric group comparisons were retained for the composite indices on the basis of their approximately symmetric distributions and the robustness of the Welch test to moderate departures from normality, while rank-based methods were used for all correlation analyses.

Welch (unequal-variance) *t*-tests were used to compare composite indices between smoking and non-smoking groups. Cohen’s d was calculated as a measure of effect size. To test our secondary hypothesis, multivariable linear regression models (for birth weight—BW) and multivariable logistic regression models (for PTB and LBW) were fitted using each of the six composite indices as primary independent variables. All models were adjusted for maternal age, delivery mode, and oxytocin use. Sensitivity analyses were performed in the full cohort (*n* = 66) by adding neonatal sex to the covariate set of every multivariable model. Because the number of events was limited (16 preterm births, 13 low-birth-weight infants), these sex-adjusted models are reported as sensitivity analyses rather than as primary models (Supplementary Tables S1 and S2, Supplementary Figure S1). The Benjamini–Hochberg false discovery rate (FDR) correction was applied independently within pre-specified test families—index comparisons (6 tests), regressions separately per outcome (6 tests each), and all-cohort correlations (9 tests)—to control the expected proportion of false positives among rejected hypotheses at the 5% level.

All analyses were performed using R software version 4.3.2. A two-sided significance level of p<0.05 was established.

## 3. Results

### 3.1. Clinical and Demographic Characteristics

The primary study cohort consisted of 66 dyads: 24 active smokers and 42 non-smokers. Demographic and clinical characteristics of the study cohorts are summarized in Table 1. Males accounted for 15 of 24 tobacco-exposed (62.5%) and 23 of 42 unexposed dyads (54.8%; *p* = 0.541).

Preterm birth occurred in 9 of 24 tobacco-exposed pregnancies (37.5%) compared with 7 of 42 unexposed pregnancies (16.7%; unadjusted odds ratio 3.00, Fisher exact *p* = 0.076), and low birth weight in 8 of 24 (33.3%) compared with 5 of 42 (11.9%; unadjusted odds ratio 3.70, Fisher exact *p* = 0.053). Mean gestational age was 36.40 ± 4.74 weeks in exposed versus 38.20 ± 3.14 weeks in unexposed pregnancies (*p* = 0.104) and mean birth weight 2827.9 ± 944.3 g versus 3141.7 ± 743.6 g (*p* = 0.170); Apgar scores did not differ at 1 or 5 min (*p* = 0.988 and *p* = 0.851). Both binary outcome estimates are consistent in direction and magnitude with the established association between gestational tobacco exposure and adverse birth outcomes, but neither reached statistical significance in this cohort (Supplementary Table S5).

### 3.2. Primary Hypothesis: Composite Stress Pathway Associations by Tobacco Exposure

**Figure 1:**
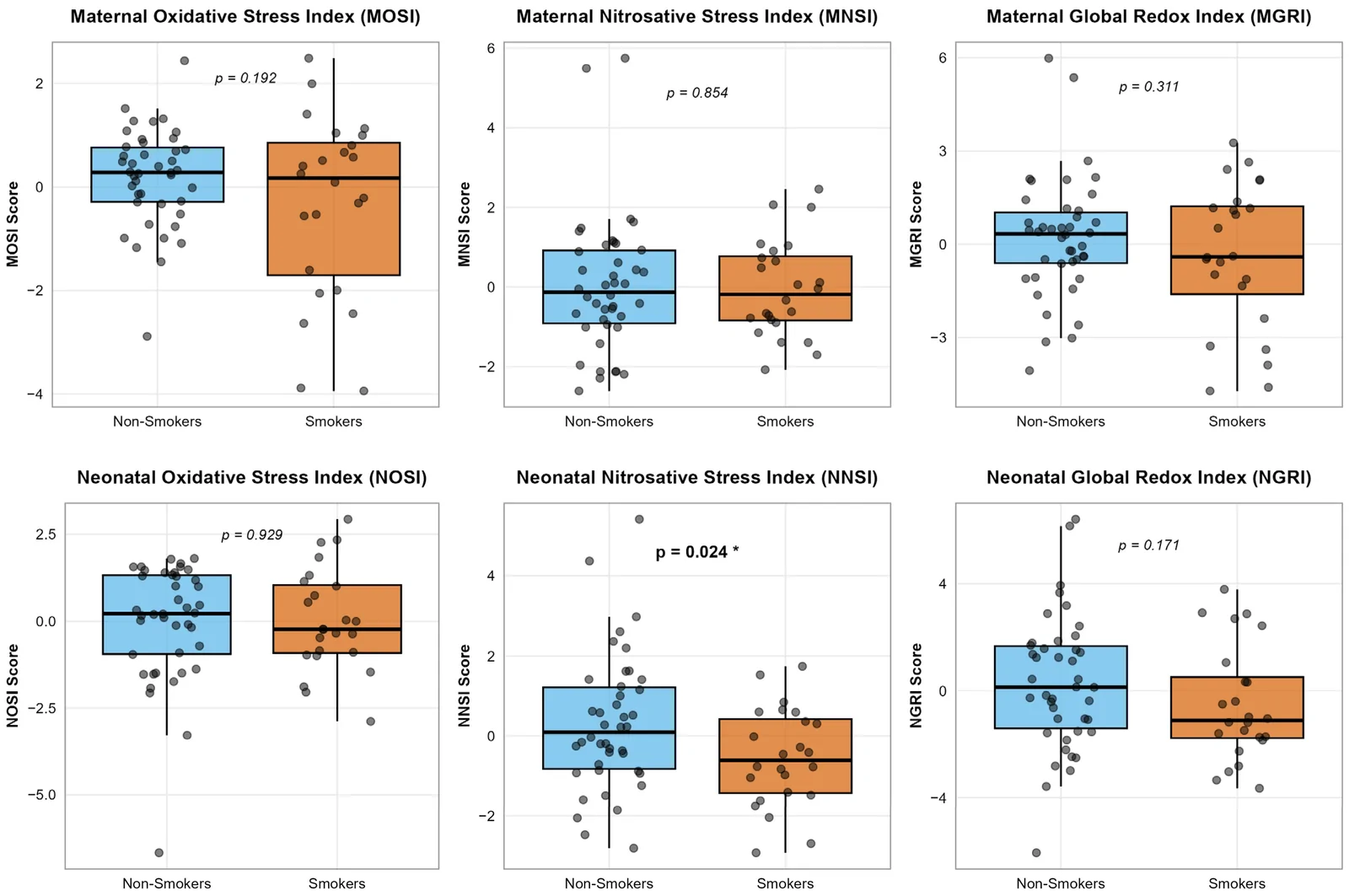
Comparison of maternal and neonatal composite stress indices (OSI, NSI, GRI) between gestational tobacco exposure groups. Boxplots show raw data jitter, medians, and IQR (Interquartile Range), annotated with nominal and FDR adjusted *p*-values. * *p* < 0.05.

Pathway-specific composite indices (OSI, NSI, GRI) were compared between the smoking and non-smoking cohorts in both compartments separately (Table 2). In the maternal compartment, active smoking during pregnancy was not associated with any significant alterations in maternal OSI (MOSI; p=0.192), maternal NSI (MNSI; p=0.854), or maternal GRI (MGRI; p=0.311). In the neonatal compartment, the Neonatal NSI (NNSI) was nominally significantly lower in the smoking cohort compared to the non-smoking cohort (Mean: −0.536 vs. 0.306; Mean Difference = −0.842; 95% CI: −1.567 to −0.117; p=0.024; Cohen’s d=−0.545). No significant differences were observed for Neonatal OSI (NOSI; p=0.929) or Neonatal GRI (NGRI; p=0.171). The lower NNSI observed in tobacco-exposed neonates was nominally significant (*p* = 0.024) but did not remain significant after false-discovery-rate correction within the family of six composite-index comparisons (adjusted *p* = 0.142) (Supplementary Table S4, Supplementary Figure S2). These primary cohort comparisons are visualized in Figure 1.

### 3.3. Secondary Hypothesis: Associations of Composite Indices with Clinical Outcomes

To evaluate the clinical impact of these composite stress pathways, multivariable regression models were fitted to predict Birth Weight, PTB, and LBW using each composite index as a primary predictor, adjusting for maternal age, delivery mode, and oxytocin use (Table 3).

Several composite stress indices emerged as highly significant independent predictors of birth outcomes.

Maternal Global Redox Index (MGRI): Higher maternal global redox score was strongly associated with increased birth weight (β=157.1 g per score unit; p<0.001) and lower risks of preterm birth (adjusted OR [aOR] = 0.35; 95% CI: 0.18 to 0.57; p<0.001) and low birth weight (aOR = 0.47; 95% CI: 0.29 to 0.70; p<0.001).

Maternal Oxidative Stress Index (MOSI)**:** Higher MOSI predicted higher birth weight (β=267.3 g; p<0.001) and lower odds of preterm birth (aOR = 0.31; p<0.001).

Maternal Nitrosative Stress Index (MNSI)**:** Higher MNSI was associated with significantly lower odds of preterm birth (aOR = 0.37; p=0.005) and low birth weight (aOR = 0.41; p=0.016).

Neonatal Nitrosative Stress Index (NNSI)**:** Higher NNSI (i.e., normal/ non depleted cord blood nitrosative metabolites) was a significant predictor of higher birth weight (β=151.8 g; p=0.016), lower odds of preterm birth (aOR = 0.52; 95% CI: 0.29 to 0.84; p=0.014), and lower odds of low birth weight (aOR = 0.53; p=0.026).

**Figure 2:**
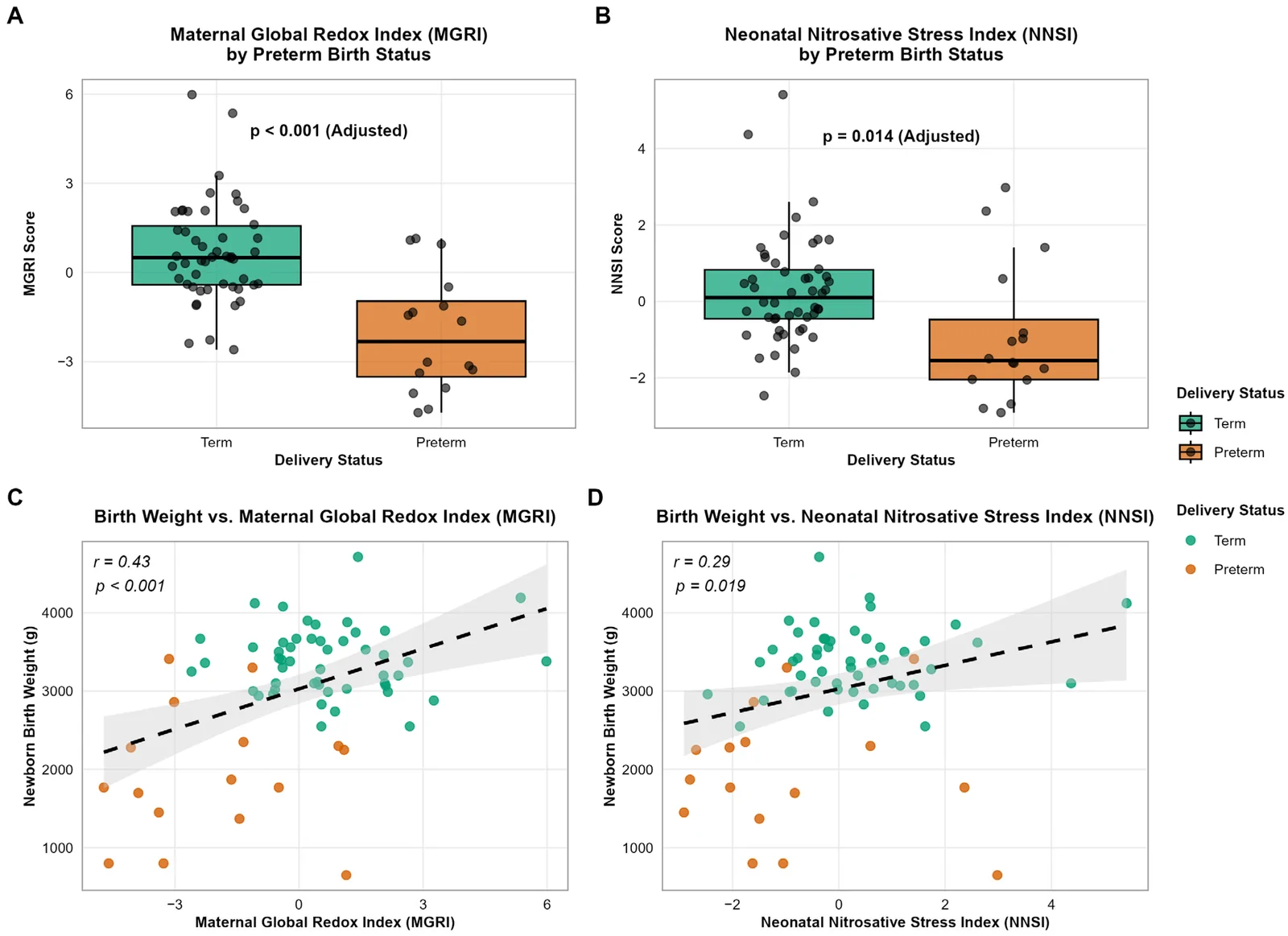
Associations of maternal and neonatal composite stress indices with adverse clinical outcomes. Panels (**A**,**B**) show boxplots of MGRI and NNSI by preterm birth status, showing significant index depletion in preterm cases. Panels (**C**,**D**) show scatterplots with regression lines for newborn birth weight vs. MGRI and NNSI, colored by preterm delivery status.

All associations between composite indices and clinical outcomes that were significant in unadjusted analyses remained significant after false-discovery-rate correction within each outcome family (all adjusted *p* ≤ 0.040; Supplementary Table S4). Adding neonatal sex to all multivariable models left every association materially unchanged, and neonatal sex was not an independent predictor of birth weight, preterm birth, or low birth weight in any model (Supplementary Tables S1 and S2, Supplementary Figure S1). These clinical associations are visualized in Figure 2.

Because the exposure subgroups were small (24 exposed and 42 unexposed dyads, with 16 preterm births and 13 low-birth-weight infants in total), stratified index–outcome analyses were pre-specified as exploratory. They are presented in the Supplementary Materials without covariate adjustment and without a formal test for interaction and are interpreted as hypothesis-generating rather than confirmatory (Supplementary Table S6, Supplementary Figure S3).

### 3.4. Compartmental Correlations Stratified by Gestational Tobacco Exposure

To examine the physiological connection between the maternal peripheral circulation and the neonatal umbilical cord circulation, Spearman correlation analyses were conducted for all individual biomarkers and composite stress indices. Analyses were performed across the entire cohort and stratified by gestational tobacco exposure (Table 4). Spearman rank correlation was used for these compartmental analyses because several individual biomarker distributions departed from normality (Supplementary Table S3), and rank-based correlation is robust to non-normality and outliers.

A significant positive correlation was observed between maternal and neonatal nitrite levels in the overall cohort (ρ=0.420, p<0.001). When stratified, this correlation was moderate in non-smokers (ρ=0.319, p=0.040) but became markedly stronger and highly significant in active smokers (ρ=0.610, p=0.002). This suggests enhanced maternal-fetal synchronization of nitrosative status under tobacco-induced stress.

A strong negative correlation between maternal and neonatal GSSG was observed in the overall cohort (ρ=−0.452, p<0.001). This negative correlation was present in non-smokers (ρ=−0.337, p=0.029) and became significantly more pronounced in active smokers (ρ=−0.600, p=0.002).

In line with individual nitrite patterns, the Maternal (M) and Neonatal (N) Nitrosative Stress Indices (MNSI vs. NNSI) were positively correlated in the overall cohort (ρ=0.274, p=0.026) and showed consistent positive trends in both non-smokers (ρ=0.264, p=0.092) and smokers (ρ=0.344, p=0.099). * Other biomarkers (nitrate, MDA, GSH, and the GSH/GSSG ratio) and the Oxidative and Global Redox Indices (OSI and GRI) did not show significant maternal-neonatal correlations in either group. After false-discovery-rate correction within the family of nine all-cohort compartmental correlations, the maternal–neonatal correlations for nitrite (adjusted *p* = 0.002) and GSSG (adjusted *p* = 0.001) remained significant, whereas the MNSI–NNSI correlation did not (adjusted *p* = 0.078; Supplementary Table S4).

### 3.5. Exploratory Supplementary Analyses

Supplementary component-level analyses were conducted to explore individual components to further analyze NNSI related findings. The results revealed a lower neonatal nitrite concentration with 29.9% in smokers, than non-smokers (*p* = 0.029), whereas neonatal nitrate showed a smaller, non-significant 12.4% difference (*p* = 0.264). Regarding clinical outcomes, both maternal and neonatal nitrite were significantly associated with birth weight, preterm birth, and low birth weight, and these nitrite-specific associations all survived Benjamini–Hochberg FDR correction. In contrast, nitrate concentrations were not significantly associated with any of the birth outcomes. To determine whether NNSI remained associated with birth outcomes after accounting for smoking status, we fitted exploratory nested models comparing a base specification (smoking + clinical covariates) with an expanded specification adding NNSI. For birth weight, NNSI remained nominally associated after adjustment for smoking (β = 132.2 g, *p* = 0.043). For preterm birth, the smoking-adjusted NNSI odds ratio was 0.557 (*p* = 0.034). For low birth weight, the association was borderline (OR = 0.577, *p* = 0.076). Limited case numbers prevent robust formal analyses, the findings remaining strictly exploratory. All the results are presented in the Supplementary Tables S5–S10.

## 4. Discussion

The present study examined the associations between gestational tobacco exposure and perinatal oxidative and nitrosative stress profiles using composite pathway-specific indices (OSI, NSI, GRI) assessed separately in maternal and neonatal blood compartments. The primary hypothesis posited that active gestational smoking would be associated with elevated maternal and neonatal composite oxidative stress indices (MOSI and NOSI). The secondary hypothesis posited that these composite stress indices would be associated with clinical outcomes, specifically birth weight and preterm birth.

The principal findings of this study revealed that active gestational smoking appears to be associated with a significant depletion of the Neonatal Nitrosative Stress Index (NNSI) in umbilical cord blood, a finding that did not support the primary hypothesis of increased oxidative stress but instead revealed a potentially important nitrosative depletion. Meanwhile both maternal and neonatal composite stress indices (particularly MGRI and NNSI) emerged as significant independent predictors of low birth weight, and preterm birth in multivariable models.

### 4.1. Neonatal Nitrosative Stress Depletion

The observation that NNSI was nominally significantly lower in the tobacco-exposed group may reflect a clinically relevant biochemical perturbation in the neonatal compartment. Nitrosative status, as reflected by nitrite and nitrate levels, is considered a major reservoir of nitric oxide bioavailability in the fetoplacental circulation. NO is a critical regulator of vascular tone in the umbilical cord, which lacks autonomic innervation and therefore relies heavily on local endothelial mediators for blood flow regulation [15,37].

Several lines of evidence suggest plausible mechanisms through which gestational tobacco exposure may contribute to reduced neonatal NO metabolite levels. An earlier study by Andersen et al. reported a 40% reduction in eNOS activity (*p* = 0.006) and a 32% reduction in eNOS concentration (*p* = 0.053) in fetal umbilical veins exposed to maternal smoking [38]. Zahorán et al. also demonstrated NOS3 deactivation, increased ROS formation, and compensatory upregulation of the NOS2 pathway in the context of smoking, with alterations that were vessel-specific and proportional to the level of tobacco exposure [15]. Additionally, Ulm et al. reported that umbilical arteries of neonates born to smoking mothers contained significantly less L-arginine and L-citrulline, which are the substrate and co-products of NOS-mediated NO synthesis, compared with non-smoking controls [39]. Dugmonits et al. described arginase upregulation and potential NOS3 uncoupling in fetal red blood cells exposed to maternal smoking, which may further divert L-arginine away from NO production and toward superoxide generation [40].

In the maternal compartment, Chełchowska et al. reported that serum NO, eNOS, and total antioxidant capacity (TAC) were significantly lower in smoking pregnant women, while iNOS and total oxidant capacity (TOC) were elevated, with multivariate regression confirming independent associations between NO concentration and both eNOS activity and cotinine levels [22]. These findings collectively suggest that tobacco smoke constituents may impair NO biosynthesis at multiple levels from substrate availability to enzyme expression and activity, in both maternal and fetal vascular beds.

The NNSI depletion observed in the present study is broadly consistent with these mechanisms, although the composite nature of the index, which aggregates standardized nitrite and nitrate Z-scores, precludes direct mechanistic inference regarding specific enzymatic pathways. Nitrite and nitrate were combined within a single nitrosative index because they rep-resent sequential products of nitric oxide oxidation and together approximate total nitric oxide metabolite availability; nitrite is the more labile, biologically active reservoir, whereas nitrate is the more stable terminal metabolite, and the two therefore may carry complementary information. Their divergent compartmental behavior, a robust maternal–neonatal correlation for nitrite but not for nitrate, is a hint for this interpretation.

### 4.2. Neonatal Nitrosative Depletion and Clinical Outcomes

In the secondary outcome analysis, lower NNSI was associated with lower birth weight (β = 151.8 g per unit, *p* = 0.016) and higher odds of preterm birth (aOR = 0.52, *p* = 0.014). These outcome associations withstood false-discovery-rate correction (adjusted *p* ≤ 0.040), whereas the group difference in NNSI itself did not; the clinical associations are therefore the more robust element of these findings. While these associations do not establish a causal pathway, they are consistent with the hypothesis that reduced NO bioavailability in umbilical circulation may contribute to increased placental vascular resistance and impaired fetoplacental perfusion. Nitrite has been shown to serve as an important reservoir of NO bioactivity within the placenta, with nitrite-mediated vasorelaxation in human chorionic plate vessels being enhanced under hypoxic conditions and dependent on the NO-sGC-cGMP (Nitric oxide-soluble guanylyl cyclase-cyclic guanosine monophosphate) pathway [37]. This mechanism may be particularly relevant in pregnancies complicated by tobacco exposure, where chronic hypoxia and endothelial dysfunction may compromise oxygen-dependent NOS activity, making nitrite reduction to NO a critical compensatory pathway [15,37].

A recent systematic review and meta-analysis of 48 studies (4684 participants) examining oxidative stress biomarkers in fetal growth restriction (FGR) reported that cord blood MDA levels were significantly elevated (Cohen’s d = 0.37, *p* = 0.01) and SOD activity was markedly reduced (Cohen’s d = −1.98, *p* < 0.001) in FGR compared with normal pregnancies, whereas differences in total antioxidant capacity, GSH, GPx, and catalase did not reach statistical significance [41]. The discrepancy between these findings and the present NNSI results may reflect differences in study populations (FGR of mixed etiology vs. tobacco-specific exposure), the composite nature of the NNSI, or the specific contribution of tobacco-induced eNOS suppression that may not be captured in heterogeneous FGR cohorts.

It should be acknowledged that the observed associations may also reflect confounding by unmeasured variables or reverse causation, whereby adverse pregnancy conditions themselves alter neonatal redox profiles. The cross-sectional nature of the biomarker assessment at delivery limits the ability to determine the temporal sequence of these associations.

### 4.3. Maternal Composite Indices: Absence of Group Differences Yet Strong Clinical Predictive Value

In the maternal compartment, composite indices did not differ significantly between smokers and non-smokers (MOSI *p* = 0.192; MNSI *p* = 0.854; MGRI *p* = 0.311). This observation may suggest that maternal systemic homeostasis partially buffers or compensates for the direct chemical insults of tobacco smoke, possibly through upregulation of antioxidant defense mechanisms. Chełchowska et al. reported that while smoking mothers exhibited elevated total oxidant capacity and reduced GSH/GSSG ratios, compensatory increases in certain antioxidant parameters were also observed [21]. Alternatively, the lack of significant maternal differences may reflect insufficient statistical power given the sample size of 66 dyads, or the possibility that placental tissue bears the primary burden of tobacco-induced oxidative damage, and not maternal systemic circulation. Hoch et al. demonstrated an 80% increase in DNA breaks and shortened telomeres in first-trimester placentas of verified smokers, alongside paradoxically reduced ROS-mediated DNA (deoxyribonucleic acid) damage and absent upregulation of antioxidant defense machinery, suggesting a delay in the establishment of physiological uteroplacental blood flow [19]. Suter and Aagaard similarly reported that placentas from smoking mothers exhibited increased markers of lipid peroxidation, elevated CYP1A1 staining, and decreased total antioxidant capacity, with a shift in the oxidative/antioxidative balance toward the oxidative side [10].

Despite the absence of significant group differences, maternal composite indices emerged as strong predictors of obstetric outcomes in the pooled cohort. Lower MGRI was associated with lower birth weight (β = 157.1 g, *p* < 0.001) and a higher risk of preterm birth (aOR = 0.35, *p* < 0.001, indicating a nearly 3-fold increase in the odds of PTB for every unit decrease in MGRI). Similarly, lower Maternal Oxidative Stress Index (MOSI) and Nitrosative Stress Index (MNSI) were significant predictors of adverse birth outcomes. Elevated maternal oxidative stress during pregnancy has been previously associated with restricted fetal growth, gestational hypertension, and preeclampsia in numerous studies [27,28,29]. Eick et al. reported that urinary oxidative stress biomarkers, particularly F2-isoprostane metabolites, were associated with increased odds of preterm birth in a large multi-cohort analysis (*n* = 1916) [42]. Baghsheikhi et al. demonstrated in the TIDES cohort study (*n* = 561) that early pregnancy oxidative stress biomarkers, including MDA, F2-isoprostanes, 8-OHdG (8-hydroxy-2′-deoxyguanosine), and dityrosine, were persistently associated with reduced fetal growth velocities across both the second and third trimesters, with first-trimester lipid peroxidation markers showing the strongest associations with third trimester estimated fetal weight velocity [43].

The present findings suggest that while maternal systemic redox status may not be directly affected by tobacco exposure in a manner detectable by these composite indices, it may nonetheless serve as a sensitive surrogate marker for underlying placental or gestational pathologies that are associated with preterm delivery and fetal growth restriction. In exploratory stratified analyses, associations between composite indices and birth weight appeared more pronounced within the tobacco-exposed stratum (Supplementary Table S10); given the small stratum sizes and the absence of a formal test for interaction, this pattern should be regarded as a hypothesis for future adequately powered studies.

### 4.4. Compartmental Correlations and Placental Transfer

The nominally significant positive correlation between maternal and neonatal NSI (Spearman ρ = 0.274, *p* = 0.026; FDR-adjusted *p* = 0.078), in the absence of significant correlations for OSI or GRI, may suggest that nitrosative metabolites traverse the placental barrier more readily than oxidative stress markers, or that maternal and fetal nitrosative pathways share common regulatory mechanisms. This finding is consistent with experimental evidence from Schroeder et al., who demonstrated in a sheep model that the placental permeability-surface product for nitrite was many-fold higher than that reported for chloride anions, suggesting a transfer mechanism that is not exclusively dependent on simple diffusion [33]. Von Mandach et al. reported that NO metabolite levels were higher in fetal compartments than in co-sampled maternal plasma, and that levels increased through normal and particularly abnormal pregnancy, predominantly in the fetal compartments [32]. Tropea et al. further demonstrated that nitrite serves as an important reservoir of NO bioactivity within the placenta, with nitrite-mediated vasorelaxation being enhanced under hypoxic conditions [37].

However, the modest magnitude of the maternal–neonatal NSI correlation warrants cautious interpretation, and the possibility of shared confounders influencing both compartments cannot be excluded. The absence of significant correlations for OSI and GRI may reflect the selective barrier function of the placenta, which may differentially modulate the transfer of lipid peroxidation products and glutathione species compared with small inorganic anions such as nitrite and nitrate. The consistent inverse maternal–neonatal correlation for oxidized glutathione, observed in both smokers and non-smokers and the single strongest correlation after false-discovery-rate correction (adjusted *p* = 0.001), may indicate compartment-specific redox handling, hinting a more complex mechanism than passive equilibration across the placenta and merits mechanistic study.

### 4.5. Composite Indices in the Context of Existing Oxidative Stress Scoring Systems

The composite indices employed in this study share conceptual similarities with previously validated scoring systems, particularly the OXY-SCORE developed by Veglia et al. [20,21]. The OXY-SCORE integrates plasma MDA, GSSG/GSH ratio, urinary isoprostanes, GSH, tocopherols, and individual antioxidant capacity into a single standardized score computed by subtracting a protection score from a damage score. Sánchez-Rodríguez and Mendoza-Núñez, in a comprehensive review of 490 articles on oxidative stress indices, concluded that the OSI, OXY-SCORE, and OSS have demonstrated reliability and clinical utility across diverse conditions, including cardiovascular disease, aging, and recurrent pregnancy loss [31]. The present study extends this approach by: (1) separating oxidative and nitrosative pathways into distinct indices (OSI and NSI), (2) applying these indices simultaneously to both maternal and neonatal compartments, and (3) evaluating their associations with perinatal clinical outcomes. However, the composite indices used here have not been externally validated, and the equal weighting of Z-score components represents a simplifying assumption that may not reflect the true biological contribution of each biomarker to clinical outcomes. NNSI combines the correlated nitrite and nitrate measurements into a single standardized index, reducing multiple testing and providing a compartment-level measure associated with birth weight and preterm birth after false-discovery-rate correction. The group-level difference in NNSI by exposure status was not significant after FDR adjustment, therefore its incremental value over single biomarkers remains to be demonstrated in larger cohorts.

### 4.6. Strengths

This study has several methodological strengths. The prospective design with simultaneous assessment of both maternal and neonatal compartments enables compartment-specific evaluation of redox perturbations. The use of composite pathway-specific indices, rather than individual biomarkers alone, is consistent with AHA recommendations for measuring multiple independent indices of oxidative stress in human studies. The conversion of all biomarker values to SI units (μmol/L) facilitates comparison with other studies and aligns with AHA guidance to report values in absolute molar concentrations. The adjustment for relevant clinical covariates (maternal age, delivery mode, oxytocin use) in multivariable models strengthens the internal validity of the secondary outcome analyses.

### 4.7. Limitations

Several important limitations should be acknowledged. The sample size of 66 dyads restricts statistical power, particularly for subgroup analyses and clinical regression models, and increases the risk of both type I and type II errors; it also limits the number of covariates that can be reliably included in multivariable models without overfitting. Because multiple composite indices were tested in parallel, false-discovery-rate correction was applied within pre-defined families; the group difference in NNSI and the MNSI–NNSI correlation did not survive this correction and are consequently presented as hypothesis-generating rather than confirmatory.

Maternal smoking status was defined by self-report at admission without biochemical validation. Self-reported smoking during pregnancy is subject to substantial misclassification. Ashford et al. reported misclassification rates of 26–35% across trimesters when self-report was compared with urinary cotinine validation, and Shipton et al. estimated that reliance on self-report underestimated true smoking prevalence by 25% in a Scottish population-based study [44,45]. This misclassification may attenuate observed associations and does not capture dose–response relationships or passive smoke exposure. Smoking status was captured as a binary self-reported variable without intensity, duration, or trimester-specific detail; combined with single delivery-time sampling, this precludes assessment of whether biomarkers reflect recent versus cumulative exposure and may misclassify women who ceased smoking earlier in pregnancy. Cotinine or comparable biochemical verification was not available, so the dose–response and trimester-specific analyses requested for this cohort cannot be performed.

Although all multivariable models could be adjusted for neonatal sex, the limited number of clinical events (16 preterm births and 13 low-birth-weight infants) constrains the number of covariates that can be included without risk of overfitting; sex-adjusted models are therefore reported as sensitivity analyses. The observational design also precludes causal inference, and residual confounding by unmeasured variables, such as maternal nutrition, socioeconomic status, body mass index, parity, and environmental co-exposures, cannot be excluded.

Biomarker assessment at a single time point (delivery) does not capture the dynamic trajectory of oxidative and nitrosative stress throughout gestation, which may be particularly relevant given evidence from the TIDES cohort that trimester-specific associations between oxidative stress and fetal growth may differ substantially. Because all samples were obtained at delivery, the measured redox and nitric-oxide metabolite levels may be influenced by the physiological adaptations of parturition, maternal exertion during labor, and intrapartum analgesia, which can independently alter oxidative and nitrosative status; delivery-time sampling therefore reflects a peri-partum snapshot rather than gestational exposure over time. Data on mode and duration of intrapartum analgesia and on length of labor were not recorded in the study database and could not be added retrospectively, so these influences could not be quantified. The composite indices have not been externally validated either, and the equal weighting of Z-score components represents a simplifying assumption; future studies should consider data-driven weighting approaches and external validation in independent cohorts [43].

The single-center design may limit the generalizability of these findings to other populations and clinical settings.

## 5. Conclusions

In this prospective cohort, gestational tobacco exposure was associated with lower neonatal nitrosative-stress markers, reflected by NNSI. However, this finding did not remain significant after false-discovery-rate correction and should be considered hypothesis-generating. Lower maternal and neonatal redox indices, particularly MGRI and NNSI, were associated with lower birth weight and greater odds of preterm birth independent of exposure status; these associations remained after multiple-testing correction and adjustment for neonatal sex.

These composite indices may provide an integrated measure of perinatal redox status but require validation in larger cohorts with biochemically verified tobacco exposure before clinical use.

## Figures and Tables

**Table 1:** Clinical and demographic characteristics of maternal–neonatal dyads by gestational tobacco exposure.

Parameter	Non-Smokers (*n* = 42)	Smokers (*n* = 24)	*p*-Value
Maternal Age (years)	26.3 ± 6.5	26.4 ± 6.3	0.952
Gestational Age (weeks)	38.2 ± 3.1	36.4 ± 4.7	0.067
Birth Weight (g)	3142 ± 744	2828 ± 944	0.125
**Delivery Mode**			
Cesarean Section, *n* (%)	10 (23.8%)	4 (16.7%)	0.499
Vaginal Delivery, *n* (%)	32 (76.2%)	20 (83.3%)	
Oxytocin Use, *n* (%)	12 (28.6%)	6 (25.0%)	0.753
Preterm Birth (PTB), *n* (%)	7 (16.7%)	9 (37.5%)	0.058
Low Birth Weight (LBW), *n* (%)	5 (11.9%)	8 (33.3%)	**0.035**
**Newborn Sex**			
Male, *n* (%)	23 (54.8%)	15 (62.5%)	0.541
Female, *n* (%)	19 (45.2%)	9 (37.5%)	

**Table 2:** Compartment-specific composite stress index comparisons (Smokers vs. Non-Smokers).

Compartment/Composite Index	Smokers Mean	Non-Smokers Mean	Mean Difference (95% CI)	Cohen’s d	*p*-Value	Adjusted *p*-Value
**Maternal Compartment**						
OSI (MOSI)	−0.327	0.187	−0.513 (−1.298, 0.272)	−0.325	0.192	0.384
NSI (MNSI)	−0.043	0.025	−0.068 (−0.799, 0.664)	−0.046	0.854	0.929
GRI (MGRI)	−0.370	0.211	−0.581 (−1.725, 0.563)	−0.252	0.311	0.499
**Neonatal Compartment**						
OSI (NOSI)	0.023	−0.013	0.035 (−0.757, 0.827)	0.022	0.929	0.929
NSI (NNSI)	−0.536	0.306	−0.842 (−1.567, −0.117)	−0.545	0.024	0.142
GRI (NGRI)	−0.513	0.293	−0.806 (−1.970, 0.358)	−0.342	0.171	0.384

**Table 3:** Multivariable regression models evaluating associations between composite stress indices and clinical outcomes.

Composite Index Term.	Birth Weight (g) [β ± SE]	Preterm Birth (PTB) [aOR (95% CI)]	Low Birth Weight (LBW) [aOR (95% CI)]
MOSI	+267.3 ± 69.7 *	0.31 (0.14, 0.56) *	0.42 (0.22, 0.72) **
MNSI	+116.2 ± 64.3	0.37 (0.17, 0.69) **	0.41 (0.18, 0.79) *
MGRI	+157.1 ± 42.5 *	0.35 (0.18, 0.57) *	0.47 (0.29, 0.70) *
NOSI	−0.8 ± 70.3	1.03 (0.68, 1.58)	0.95 (0.60, 1.50)
NNSI	+151.8 ± 61.2 *	0.52 (0.29, 0.84) *	0.53 (0.28, 0.88) *
NGRI	+78.4 ± 45.1	0.76 (0.53, 1.02)	0.72 (0.48, 1.01)

Models adjusted for maternal age, delivery mode, and oxytocin use. Coefficients (β) represent change in birth weight per unit increase in the index. Odds ratios represent adjusted odds of delivery event per unit increase in the index. Significance: * *p* < 0.05, ** *p* < 0.01, *** *p* < 0.001. All estimates flagged as significant remained significant after Benjamini–Hochberg correction applied within each outcome family (all adjusted *p* ≤ 0.040; Supplementary Table S4).

**Table 4:** Maternal–neonatal compartmental correlation analysis stratified by gestational tobacco exposure.

Biomarker/Index Pair	All Cohort (*n* = 66) Spearman r (*p*-Value)	Non-Smokers (*n* = 42) Spearman r (*p*-Value)	Smokers (*n* = 24) Spearman r (*p*-Value)
Nitrite (M vs. N)	**0.420 (<0.001)**	**0.319 (0.040)**	**0.610 (0.002)**
Nitrate (M vs. N)	0.113 (0.366)	0.180 (0.254)	−0.037 (0.865)
MDA	−0.174 (0.163)	−0.201 (0.202)	−0.163 (0.448)
GSH	0.173 (0.165)	0.230 (0.143)	0.064 (0.768)
GSSG	**−0.452 (<0.001)**	**−0.337 (0.029)**	**−0.600 (0.002)**
GSH/GSSG ratio	<0.001 (0.999)	0.132 (0.406)	−0.068 (0.753)
OSI (MOSI vs. NOSI)	−0.208 (0.095)	−0.254 (0.104)	−0.217 (0.308)
NSI (MNSI vs. NNSI)	**0.274 (0.026)**	0.264 (0.092)	0.344 (0.099)
GRI (MGRI vs. NGRI)	0.114 (0.361)	0.048 (0.764)	0.075 (0.728)

Bold values denote statistical significance (*p* < 0.05).

## Data Availability

The data presented in this study are available upon reasonable request from the corresponding author, following approval for data sharing by the Ethics Committee of the George Emil Palade University of Medicine, Pharmacy, Science, and Technology of Târgu Mureș.

## Supplementary Materials

The following supporting information can be downloaded at: https://www.mdpi.com/article/10.3390/antiox15080996/s1, Figure S1: Primary and newborn-sex-adjusted associations between composite redox indices and birth outcomes; Figure S2: Neonatal nitrite concentrations according to maternal smoking status; Figure S3: Neonatal nitrate concentrations according to maternal smoking status; Figure S4: Component-specific associations of maternal and neonatal nitrite and nitrate with birth outcomes; Table S1: Comparison of primary and newborn-sex-adjusted associations between composite redox indices and birth outcomes; Table S2: Contribution of newborn sex to multivariable models of composite redox indices and birth outcomes; Table S3: Shapiro-Wilk assessment of the distributions of redox biomarkers, composite indices, and clinical variables; Table S4: Benjamini-Hochberg false-discovery-rate correction of the original study analyses; Table S5: Exploratory smoking-group comparisons of maternal and neonatal nitrite and nitrate concentrations; Table S6: Exploratory component-level associations of maternal and neonatal nitrite and nitrate with birth weight; Table S7: Exploratory component-level associations of maternal and neonatal nitrite and nitrate with preterm birth and low birth weight; Table S8: Exploratory nested models evaluating neonatal nitrosative stress index associations after adjustment for maternal smoking; Table S9: Benjamini-Hochberg false-discovery-rate correction of the exploratory analyses; Table S10: Crude birth outcomes according to maternal tobacco exposure; Table S11: Exploratory associations between composite redox indices and birth outcomes stratified by maternal smoking status.

## Abbreviations

The following abbreviations are used in this manuscript:

8-OH-dG	8-hydroxy-2′-deoxyguanosine
AHA	American Heart Association
BW	Birth weight
CAT	Catalase
cGMP	Cyclic guanosine monophosphate
CI	Confidence Interval
DNA	Deoxyribonucleic acid
DTNB	5,5′-dithiobis (2-nitrobenzoic acid)
ECCH	Emergency County Clinical Hospital
eNOS	Endothelial Nitric Oxide Synthase
FGR	Fetal growth restriction
GEP UMPhST	G.E. Palade University of Medicine, Science, and Technology
GPx	Glutathione peroxidase
GR	Glutathione reductase
GRI	Global redox index
GSH	Reduced glutathione
GSSG	Oxidized glutathione
HPLC	High-performance liquid chromatography
iNOS	Inducible Nitric Oxide Synthase
IQR	Interquartile Range
LBW	Low birth weight
M	Maternal
MDA	Malondialdehyde
N	Neonatal
NO	Nitric Oxide
NOS	Nitric Oxide Synthase
NSI	Nitrosative Stress Index
OR	Odds ratio
OSI	Oxidative Stress Index
OSS	Oxidative Stress Score
PTB	Preterm birth
RNS	Reactive nitrogen species
ROS	Reactive oxygen species
RR	Relative risk
sGC	Soluble guanylyl cyclase
SI	International System of units
SOD	Superoxide dismutase
TAC	Total Antioxidant Capacity
TOX	Total Oxidant Capacity
TRAP	Total Radical-Trapping Antioxidant Parameters
WNT	Wingless-related Integration Site
