# Supplementary material for: Perinatal Redox Dysregulation in Gestational Tobacco Exposure: Compartment-Specific Composite Indices and Their Association with Birth Outcomes—A Prospective Cohort Study
Source: Antioxidants (Basel). 2026 Aug 11;15(8):996. doi: 10.3390/antiox15080996 (PMC13509844; doi:10.3390/antiox15080996)
Supplement: Supplementary file 1 [file antioxidants-15-00996-s001.zip › antioxidants-4457347-supplementary.pdf]

Table S1. Comparison of primary and newborn-sex-adjusted associations between composite redox indices and birth outcomes.

Index	Outcome	Measure	Primary estimate	Primary 95% CI	Primary p (fmt)	Primary FDR p (fmt)	Sex-adj estimate	Sex-adj 95% CI	Sex-adj p (fmt)	Sex-adj FDR p (fmt)	Conclusion unchanged
MOSI	Birth weight (g)	β (g)	267.5	(128.1, 406.8)	<0.001***	0.001**	272.0	(131.5, 412.5)	<0.001***	0.002**	yes
MOSI	Preterm birth	aOR	0.31	(0.16, 0.61)	<0.001***	0.002**	0.32	(0.16, 0.63)	0.001**	0.003**	yes
MOSI	Low birth weight	aOR	0.42	(0.24, 0.75)	0.004**	0.011*	0.43	(0.24, 0.77)	0.005**	0.015*	yes
MNSI	Birth weight (g)	β (g)	116.2	(-12.4, 244.9)	0.076	0.105	116.4	(-16.1, 248.8)	0.084	0.113	yes
MNSI	Preterm birth	aOR	0.37	(0.18, 0.74)	0.005**	0.010**	0.32	(0.15, 0.68)	0.003**	0.007**	yes
MNSI	Low birth weight	aOR	0.41	(0.20, 0.85)	0.016*	0.032*	0.35	(0.16, 0.76)	0.008**	0.017*	yes
MGRI	Birth weight (g)	β (g)	157.1	(72.1, 242.2)	<0.001***	0.001**	156.9	(70.8, 243.0)	<0.001***	0.002**	yes
MGRI	Preterm birth	aOR	0.35	(0.20, 0.61)	<0.001***	0.001**	0.35	(0.19, 0.62)	<0.001***	0.002**	yes
MGRI	Low birth weight	aOR	0.47	(0.30, 0.73)	<0.001***	0.005**	0.47	(0.30, 0.73)	<0.001***	0.005**	yes
NOSI	Birth weight (g)	β (g)	-0.8	(-141.3, 139.8)	0.991	0.991	-2.4	(-144.4, 139.6)	0.973	0.973	yes
NOSI	Preterm birth	aOR	1.03	(0.68, 1.55)	0.894	0.894	1.02	(0.68, 1.54)	0.930	0.930	yes
NOSI	Low birth weight	aOR	0.95	(0.61, 1.47)	0.818	0.818	0.96	(0.62, 1.48)	0.843	0.843	yes
NNSI	Birth weight (g)	β (g)	151.8	(29.5, 274.1)	0.016*	0.032*	151.0	(27.6, 274.5)	0.017*	0.035*	yes
NNSI	Preterm birth	aOR	0.52	(0.31, 0.88)	0.014*	0.021*	0.47	(0.27, 0.84)	0.010*	0.015*	yes
NNSI	Low birth weight	aOR	0.53	(0.30, 0.93)	0.026*	0.040*	0.5	(0.27, 0.90)	0.022*	0.033*	yes
NGRI	Birth weight (g)	β (g)	78.4	(-11.8, 168.7)	0.087	0.105	77.7	(-13.6, 169.0)	0.094	0.113	yes
NGRI	Preterm birth	aOR	0.76	(0.55, 1.04)	0.083	0.099	0.73	(0.52, 1.02)	0.062	0.074	yes
NGRI	Low birth weight	aOR	0.72	(0.50, 1.04)	0.078	0.094	0.71	(0.49, 1.03)	0.071	0.086	yes

Primary and newborn-sex-adjusted multivariable models estimated associations of each maternal or neonatal composite redox index with birth weight, preterm birth, and low birth weight in 66 mother-newborn pairs.

Abbreviations: beta, linear regression coefficient; aOR, adjusted odds ratio; CI, confidence interval; FDR, false discovery rate; MOSI, maternal oxidative stress index; MNSI, maternal nitrosative stress index; MGRI, maternal glutathione redox index; NOSI, neonatal oxidative stress index; NNSI, neonatal nitrosative stress index; NGRI, neonatal glutathione redox index. * $p < 0.05$, ** $p < 0.01$, and *** $p < 0.001$.

Table S2. Contribution of newborn sex to multivariable models of composite redox indices and birth outcomes.

Index	Outcome	Sex effect	Sex p	Events	Events per predictor	FDR Sex p
MOSI	Birth weight	+132.9 g	0.471			0.653
MOSI	Preterm birth	OR 1.84	0.445	16.0	3.2	0.653
MOSI	Low birth weight	OR 2.24	0.322	13.0	2.6	0.528
MNSI	Birth weight	-2.4 g	0.991			0.991
MNSI	Preterm birth	OR 4.39	0.058	16.0	3.2	0.351
MNSI	Low birth weight	OR 5.40	0.053	13.0	2.6	0.351
MGRI	Birth weight	+9.4 g	0.96			0.991
MGRI	Preterm birth	OR 3.21	0.192	16.0	3.2	0.351
MGRI	Low birth weight	OR 3.63	0.145	13.0	2.6	0.351
NOSI	Birth weight	+69.7 g	0.735			0.943
NOSI	Preterm birth	OR 2.39	0.195	16.0	3.2	0.351
NOSI	Low birth weight	OR 2.78	0.176	13.0	2.6	0.351
NNSI	Birth weight	+44.0 g	0.822			0.943
NNSI	Preterm birth	OR 3.41	0.098	16.0	3.2	0.351
NNSI	Low birth weight	OR 3.39	0.126	13.0	2.6	0.351
NGRI	Birth weight	+40.9 g	0.839			0.943
NGRI	Preterm birth	OR 2.81	0.137	16.0	3.2	0.351
NGRI	Low birth weight	OR 2.95	0.158	13.0	2.6	0.351

The table presents the estimated effect of newborn sex, its nominal and FDR-adjusted p-values, and the number of outcome events per predictor in models evaluating birth weight, preterm birth, and low birth weight.

Abbreviations: OR, odds ratio; FDR, false discovery rate; EPV, events per predictor; MOSI, maternal oxidative stress index; MNSI, maternal nitrosative stress index; MGRI, maternal glutathione redox index; NOSI, neonatal oxidative stress index; NNSI, neonatal nitrosative stress index; NGRI, neonatal glutathione redox index. Blank event and EPV cells indicate continuous-outcome linear regression models for which these measures are not applicable.

Table S3. Shapiro-Wilk assessment of the distributions of redox biomarkers, composite indices, and clinical variables.

Variable	Scale	n	Skewness	Shapiro-Wilk W	p	Normality
Nitrite, maternal	raw concentration (umol/L)	66	7.24	0.249	<0.001	REJECTED
Nitrite, maternal	natural log (as analysed)	66	1.52	0.894	<0.001	REJECTED
Nitrite, neonatal	raw concentration (umol/L)	66	5.32	0.441	<0.001	REJECTED
Nitrite, neonatal	natural log (as analysed)	66	1.18	0.923	<0.001	REJECTED
Nitrate, maternal	raw concentration (umol/L)	66	7.49	0.232	<0.001	REJECTED
Nitrate, maternal	natural log (as analysed)	66	2.88	0.776	<0.001	REJECTED
Nitrate, neonatal	raw concentration (umol/L)	66	2.38	0.77	<0.001	REJECTED
Nitrate, neonatal	natural log (as analysed)	66	0.37	0.964	0.055	not rejected
MDA, maternal	raw concentration (umol/L)	66	2.17	0.651	<0.001	REJECTED
MDA, maternal	natural log (as analysed)	66	0.58	0.938	0.003	REJECTED
MDA, neonatal	raw concentration (umol/L)	66	3.78	0.622	<0.001	REJECTED
MDA, neonatal	natural log (as analysed)	66	0.08	0.981	0.415	not rejected
GSH, maternal	raw concentration (umol/L)	66	1.17	0.928	<0.001	REJECTED
GSH, maternal	natural log (as analysed)	66	0.05	0.988	0.760	not rejected
GSH, neonatal	raw concentration (umol/L)	66	3.75	0.572	<0.001	REJECTED
GSH, neonatal	natural log (as analysed)	66	1.88	0.835	<0.001	REJECTED
GSSG, maternal	raw concentration (umol/L)	66	-0.04	0.981	0.397	not rejected
GSSG, maternal	natural log (as analysed)	66	-0.43	0.966	0.070	not rejected
GSSG, neonatal	raw concentration (umol/L)	66	3.72	0.583	<0.001	REJECTED
GSSG, neonatal	natural log (as analysed)	66	0.62	0.767	<0.001	REJECTED
MOSI	composite index	66	-1.01	0.928	<0.001	REJECTED
MNSI	composite index	66	1.26	0.91	<0.001	REJECTED
MGRI	composite index	66	0.01	0.973	0.151	not rejected
NOSI	composite index	66	-1.18	0.928	<0.001	REJECTED

Variable	Scale	n	Skewness	Shapiro-Wilk W	p	Normality
NNSI	composite index	66	0.79	0.962	0.040	REJECTED
NGRI	composite index	66	0.35	0.982	0.448	not rejected
Maternal age (years)	clinical variable	66	0.07	0.973	0.155	not rejected
Gestational age (weeks)	clinical variable	66	-1.9	0.74	<0.001	REJECTED
Birth weight (g)	clinical variable	66	-1.08	0.908	<0.001	REJECTED
Apgar, 1 min	clinical variable	66	-2.19	0.727	<0.001	REJECTED
Apgar, 5 min	clinical variable	66	-2.94	0.6	<0.001	REJECTED

Distributional normality was evaluated for raw and natural-log-transformed biomarker concentrations, composite redox indices, and continuous clinical variables in the complete study sample.

Abbreviations: MDA, malondialdehyde; GSH, reduced glutathione; GSSG, oxidized glutathione; MOSI, maternal oxidative stress index; MNSI, maternal nitrosative stress index; MGRI, maternal glutathione redox index; NOSI, neonatal oxidative stress index; NNSI, neonatal nitrosative stress index; NGRI, neonatal glutathione redox index. Normality was rejected when $p < 0.05$.

Table S4. Benjamini-Hochberg false-discovery-rate correction of the original study analyses.

Family	Test	Raw p	BH-adjusted p	Raw sig	Sig after FDR	Status change
A. Table 2 index comparisons (6 tests)	MOSI: smokers vs non-smokers	0.192	0.384	no	no	
A. Table 2 index comparisons (6 tests)	MNSI: smokers vs non-smokers	0.854	0.929	no	no	
A. Table 2 index comparisons (6 tests)	MGRI: smokers vs non-smokers	0.311	0.466	no	no	
A. Table 2 index comparisons (6 tests)	NOSI: smokers vs non-smokers	0.929	0.929	no	no	
A. Table 2 index comparisons (6 tests)	NNSI: smokers vs non-smokers	0.024	0.142	yes	no	*** LOSES significance ***
A. Table 2 index comparisons (6 tests)	NGRI: smokers vs non-smokers	0.171	0.384	no	no	
B. Table 3, Birth weight (6 tests)	MOSI -> Birth weight	<0.001	0.001	yes	yes	
B. Table 3, Birth weight (6 tests)	MNSI -> Birth weight	0.076	0.105	no	no	
B. Table 3, Birth weight (6 tests)	MGRI -> Birth weight	<0.001	0.001	yes	yes	
B. Table 3, Birth weight (6 tests)	NOSI -> Birth weight	0.991	0.991	no	no	
B. Table 3, Birth weight (6 tests)	NNSI -> Birth weight	0.016	0.032	yes	yes	
B. Table 3, Birth weight (6 tests)	NGRI -> Birth weight	0.087	0.105	no	no	
B. Table 3, Preterm birth (6 tests)	MOSI -> Preterm birth	<0.001	0.002	yes	yes	
B. Table 3, Preterm birth (6 tests)	MNSI -> Preterm birth	0.005	0.01	yes	yes	
B. Table 3, Preterm birth (6 tests)	MGRI -> Preterm birth	<0.001	0.001	yes	yes	
B. Table 3, Preterm birth (6 tests)	NOSI -> Preterm birth	0.894	0.894	no	no	
B. Table 3, Preterm birth (6 tests)	NNSI -> Preterm birth	0.014	0.021	yes	yes	
B. Table 3, Preterm birth (6 tests)	NGRI -> Preterm birth	0.083	0.099	no	no	
B. Table 3, Low birth weight (6 tests)	MOSI -> Low birth weight	0.004	0.011	yes	yes	
B. Table 3, Low birth weight (6 tests)	MNSI -> Low birth weight	0.016	0.032	yes	yes	

Family	Test	Raw p	BH-adjusted p	Raw sig	Sig after FDR	Status change
B. Table 3, Low birth weight (6 tests)	MGRI -> Low birth weight	<0.001	0.005	yes	yes	
B. Table 3, Low birth weight (6 tests)	NOSI -> Low birth weight	0.818	0.818	no	no	
B. Table 3, Low birth weight (6 tests)	NNSI -> Low birth weight	0.026	0.04	yes	yes	
B. Table 3, Low birth weight (6 tests)	NGRI -> Low birth weight	0.078	0.094	no	no	
C. Table 4 all-cohort correlations (9 tests)	Nitrite (M vs N)	<0.001	0.002	yes	yes	
C. Table 4 all-cohort correlations (9 tests)	Nitrate (M vs N)	0.366	0.412	no	no	
C. Table 4 all-cohort correlations (9 tests)	MDA (M vs N)	0.163	0.248	no	no	
C. Table 4 all-cohort correlations (9 tests)	GSH (M vs N)	0.165	0.248	no	no	
C. Table 4 all-cohort correlations (9 tests)	GSSG (M vs N)	<0.001	0.001	yes	yes	
C. Table 4 all-cohort correlations (9 tests)	GSH/GSSG ratio (M vs N)	0.999	0.999	no	no	
C. Table 4 all-cohort correlations (9 tests)	OSI (MOSI vs NOSI)	0.095	0.213	no	no	
C. Table 4 all-cohort correlations (9 tests)	NSI (MNSI vs NNSI)	0.026	0.078	yes	no	*** LOSES significance ***
C. Table 4 all-cohort correlations (9 tests)	GRI (MGRI vs NGRI)	0.361	0.412	no	no	

Raw and Benjamini-Hochberg-adjusted p-values are reported within prespecified hypothesis families for smoking-group comparisons, index-outcome associations, and maternal-neonatal correlations.

Abbreviations: BH, Benjamini-Hochberg; FDR, false discovery rate; M, maternal; N, neonatal; MDA, malondialdehyde; GSH, reduced glutathione; GSSG, oxidized glutathione; OSI, oxidative stress index; NSI, nitrosative stress index; GRI, glutathione redox index. Statistical significance was defined as $p < 0.05$.

Table S5. Exploratory smoking-group comparisons of maternal and neonatal nitrite and nitrate concentrations.

Biomarker	n smokers	n non-smokers	Geo. mean smokers	Geo. mean non-smokers	Ratio (smk/non)	Ratio 95% CI	% difference	Raw p (linear model)	Cohen d (log scale)	Welch t p	Mann-Whitney p
Maternal nitrite	24	42	0.2501	0.2873	0.87	(0.602, 1.259)	-13.0%	0.4550343889389762	-0.192	0.4077345727829741	0.2802653773370421
Maternal nitrate	24	42	30.1856	28.3303	1.065	(0.822, 1.382)	+6.5%	0.6276818298165645	0.125	0.600489543157354	0.3647059474139883
Neonatal nitrite	24	42	0.212	0.3022	0.701	(0.511, 0.964)	-29.9%	0.0291954434304342	-0.571	0.0219531098169956	0.0592728908624869
Neonatal nitrate	24	42	24.1772	27.5901	0.876	(0.693, 1.107)	-12.4%	0.2640708048126326	-0.288	0.1976659728470565	0.3682479343303962

Geometric means, smoker-to-non-smoker ratios, effect sizes, and results from parametric and nonparametric comparisons are reported separately for maternal and neonatal nitrite and nitrate.

Abbreviations: CI, confidence interval; smk/non, smokers relative to non-smokers; Geo. mean, geometric mean. Cohen's d was calculated on the natural-log scale; raw p-values were not adjusted for multiple comparisons in this table.

Table S6. Exploratory component-level associations of maternal and neonatal nitrite and nitrate with birth weight.

Biomarker	Model	n	β per ln-unit	SE	95% CI	p	β per doubling (g)	95% CI per doubling	Adj. R^2	AIC
Maternal nitrite	Base	66	358.0	139.2	(79.7, 636.3)	0.0125511446626354	248.1	(55.2, 441.0)	0.177	1066.3
Maternal nitrite	Smoking-adjusted	66	330.9	139.4	(52.1, 609.7)	0.0208203973267266	229.4	(36.1, 422.6)	0.19	1066.1
Maternal nitrate	Base	66	67.6	196.3	(-325.0, 460.2)	0.7317112514884696	46.9	(-225.3, 319.0)	0.09	1073.0
Maternal nitrate	Smoking-adjusted	66	85.6	193.7	(-301.8, 473.0)	0.6601315294153233	59.3	(-209.2, 327.8)	0.117	1071.9
Neonatal nitrite	Base	66	462.3	144.5	(173.3, 751.2)	0.0021859184637009	320.4	(120.2, 520.7)	0.219	1062.8
Neonatal nitrite	Smoking-adjusted	66	423.2	151.1	(121.0, 725.4)	0.0068417338061035	293.3	(83.9, 502.8)	0.217	1064.0
Neonatal nitrate	Base	66	172.0	226.7	(-281.3, 625.2)	0.4509539251571334	119.2	(-195.0, 433.3)	0.097	1072.5
Neonatal nitrate	Smoking-adjusted	66	110.0	227.5	(-345.0, 565.0)	0.6303954840222126	76.3	(-239.1, 391.6)	0.118	1071.8

Base and maternal-smoking-adjusted linear regression models estimated birth-weight differences per one-unit increase in the natural logarithm of each biomarker and per doubling of its concentration.

Abbreviations: beta, linear regression coefficient in grams; SE, standard error; CI, confidence interval; adjusted R-squared, coefficient of determination adjusted for model complexity; AIC, Akaike information criterion; ln-unit, one-unit increase on the natural-log scale. Estimates per doubling equal the ln-unit coefficients multiplied by ln(2).

Table S7. Exploratory component-level associations of maternal and neonatal nitrite and nitrate with preterm birth and low birth weight.

Outcome	Biomarker	Model	n	Events	EPV	aOR per ln-unit	95% CI	p	aOR per doubling	95% CI per doubling	AIC	Converged	Separation
Preterm birth	Maternal nitrite	Base	66	16	4.0	0.032	(0.004, 0.240)	0.0008000319088304	0.093	(0.023, 0.372)	57.6	yes	none detected
Preterm birth	Maternal nitrite	Smoking-adjusted	66	16	3.2	0.029	(0.004, 0.242)	0.0010549460207851	0.086	(0.020, 0.374)	55.9	yes	none detected
Preterm birth	Maternal nitrate	Base	66	16	4.0	0.445	(0.079, 2.493)	0.3567790976774667	0.57	(0.173, 1.883)	76.7	yes	none detected
Preterm birth	Maternal nitrate	Smoking-adjusted	66	16	3.2	0.32	(0.053, 1.919)	0.2124865977936192	0.454	(0.131, 1.571)	73.5	yes	none detected
Preterm birth	Neonatal nitrite	Base	66	16	4.0	0.016	(0.002, 0.165)	0.0005038698585798	0.057	(0.011, 0.287)	54.6	yes	none detected
Preterm birth	Neonatal nitrite	Smoking-adjusted	66	16	3.2	0.02	(0.002, 0.208)	0.001098811208862	0.066	(0.013, 0.337)	56.1	yes	none detected
Preterm birth	Neonatal nitrate	Base	66	16	4.0	0.75	(0.192, 2.927)	0.678458810417679	0.819	(0.319, 2.105)	77.5	yes	none detected
Preterm birth	Neonatal nitrate	Smoking-adjusted	66	16	3.2	1.046	(0.230, 4.761)	0.953135915104188	1.032	(0.361, 2.950)	75.2	yes	none detected
Low birth weight	Maternal nitrite	Base	66	13	3.2	0.077	(0.014, 0.432)	0.0035592001850388	0.17	(0.052, 0.559)	56.4	yes	none detected
Low birth weight	Maternal nitrite	Smoking-adjusted	66	13	2.6	0.054	(0.008, 0.391)	0.0038340863558825	0.133	(0.034, 0.521)	52.2	yes	none detected
Low birth weight	Maternal nitrate	Base	66	13	3.2	0.417	(0.060, 2.900)	0.3765245508638487	0.545	(0.142, 2.092)	67.7	yes	none detected
Low birth weight	Maternal nitrate	Smoking-adjusted	66	13	2.6	0.297	(0.039, 2.237)	0.2384266362078264	0.431	(0.106, 1.747)	63.2	yes	none detected
Low birth weight	Neonatal nitrite	Base	66	13	3.2	0.02	(0.002, 0.235)	0.0017805910810382	0.068	(0.012, 0.366)	50.8	yes	none detected
Low birth weight	Neonatal nitrite	Smoking-adjusted	66	13	2.6	0.027	(0.002, 0.338)	0.0050082618944911	0.083	(0.015, 0.472)	51.3	yes	none detected
Low birth weight	Neonatal nitrate	Base	66	13	3.2	0.74	(0.166, 3.305)	0.6933254132090668	0.812	(0.288, 2.290)	68.4	yes	none detected
Low birth weight	Neonatal nitrate	Smoking-adjusted	66	13	2.6	1.137	(0.204, 6.347)	0.8835639776304512	1.093	(0.332, 3.600)	64.7	yes	none detected

Base and maternal-smoking-adjusted logistic regression models estimated associations per one-unit increase in the natural logarithm of each biomarker and per doubling of its concentration, with interpretation limited by the small numbers of outcome events.

Abbreviations: aOR, adjusted odds ratio; CI, confidence interval; EPV, events per predictor; AIC, Akaike information criterion; PTB, preterm birth; LBW, low birth weight; ln-unit, one-unit increase on the natural-log scale. Estimates per doubling were derived using ln(2); absence of detected separation does not eliminate the risk of model instability arising from low EPV.

Table S8. Exploratory nested models evaluating neonatal nitrosative stress index associations after adjustment for maternal smoking.

Outcome	Model	NNSI estimate	NNSI 95% CI	NNSI p	Smoking estimate	Smoking 95% CI	Smoking p	Adj. R^2	AIC	n
Birth weight (g)	BW-0 (smoking + covariates)	-	-	-	-335.8	(-733.5, 62.0)	0.097	0.129	1070.1	66
Birth weight (g)	BW-1 (NNSI + smoking + covariates)	132.2	(4.4, 259.9)	0.043	-213.8	(-618.9, 191.4)	0.295	0.173	1067.5	66
Birth weight (g)	Model comparison (BW-1 vs BW-0)							ΔR^2=+0.044	ΔAIC=-2.5	F-test p=0.043
Preterm birth	PTB-0 (smoking + covariates)	-	-	-	OR 3.81	(1.06, 13.65)	0.04	Pseudo R^2=0.135	73.2	66 (events=16)
Preterm birth	PTB-1 (NNSI + smoking + covariates)	OR 0.557	(0.325, 0.956)	0.034	OR 2.63	(0.68, 10.15)	0.162	Pseudo R^2=0.209	69.9	66 (events=16)
Preterm birth	Comparison (PTB-1 vs -0)							Separation: M0=none, M1=none	ΔAIC=-3.3	LR-test p=0.021
Low birth weight	LBW-0 (smoking + covariates)	-	-	-	OR 5.63	(1.27, 25.00)	0.023	Pseudo R^2=0.195	62.7	66 (events=13)
Low birth weight	LBW-1 (NNSI + smoking + covariates)	OR 0.577	(0.314, 1.060)	0.076	OR 3.94	(0.84, 18.43)	0.081	Pseudo R^2=0.251	61.1	66 (events=13)
Low birth weight	Comparison (LBW-1 vs -0)							Separation: M0=none, M1=none	ΔAIC=-1.6	LR-test p=0.057

Base models containing maternal smoking and prespecified covariates were compared with expanded models additionally containing the neonatal nitrosative stress index for birth weight, preterm birth, and low birth weight.

Abbreviations: NNSI, neonatal nitrosative stress index; CI, confidence interval; OR, odds ratio; adjusted R-squared, coefficient of determination adjusted for model complexity; pseudo-R-squared, logistic-regression goodness-of-fit measure; AIC, Akaike information criterion; delta, difference between expanded and base models; LR test, likelihood-ratio test; BW, birth weight; PTB, preterm birth; LBW, low birth weight; M0, base model; M1, expanded model.

Table S9. Benjamini-Hochberg false-discovery-rate correction of the exploratory analyses.

Family	Test	Raw p	BH-adjusted p	Sig (raw)	Sig (FDR)	Status change
C2-A: Smoking-group comparisons (4 tests)	Maternal nitrite	0.455	0.607	no	no	
C2-A: Smoking-group comparisons (4 tests)	Maternal nitrate	0.628	0.628	no	no	
C2-A: Smoking-group comparisons (4 tests)	Neonatal nitrite	0.029	0.117	yes	no	*** LOSES significance ***
C2-A: Smoking-group comparisons (4 tests)	Neonatal nitrate	0.264	0.528	no	no	
C2-B: Birth weight base models (4 tests)	Maternal nitrite	0.013	0.025	yes	yes	
C2-B: Birth weight base models (4 tests)	Maternal nitrate	0.732	0.732	no	no	
C2-B: Birth weight base models (4 tests)	Neonatal nitrite	0.002	0.009	yes	yes	
C2-B: Birth weight base models (4 tests)	Neonatal nitrate	0.451	0.601	no	no	
C2-C: PTB base models (4 tests)	Maternal nitrite	<0.001	0.002	yes	yes	
C2-C: PTB base models (4 tests)	Maternal nitrate	0.357	0.476	no	no	
C2-C: PTB base models (4 tests)	Neonatal nitrite	<0.001	0.002	yes	yes	
C2-C: PTB base models (4 tests)	Neonatal nitrate	0.678	0.678	no	no	
C2-D: LBW base models (4 tests)	Maternal nitrite	0.004	0.007	yes	yes	
C2-D: LBW base models (4 tests)	Maternal nitrate	0.377	0.502	no	no	
C2-D: LBW base models (4 tests)	Neonatal nitrite	0.002	0.007	yes	yes	
C2-D: LBW base models (4 tests)	Neonatal nitrate	0.693	0.693	no	no	

Family	Test	Raw p	BH-adjusted p	Sig (raw)	Sig (FDR)	Status change
C3: NNSI smoking-adjusted (3 tests)	Birth weight (g)	0.043	0.065	yes	no	*** LOSES significance ***
C3: NNSI smoking-adjusted (3 tests)	Preterm birth	0.034	0.065	yes	no	*** LOSES significance ***
C3: NNSI smoking-adjusted (3 tests)	Low birth weight	0.076	0.076	no	no	

Raw and Benjamini-Hochberg-adjusted p-values are presented within prespecified exploratory hypothesis families covering smoking-group comparisons, component-level outcome models, and smoking-adjusted NNSI models.

Abbreviations: BH, Benjamini-Hochberg; FDR, false discovery rate; PTB, preterm birth; LBW, low birth weight; NNSI, neonatal nitrosative stress index. Statistical significance was defined as $p < 0.05$, and 'loses significance' indicates a raw p-value below 0.05 but an FDR-adjusted p-value of 0.05 or greater.

Table S10. Crude birth outcomes according to maternal tobacco exposure.

Outcome	Non-smokers (n=42)	Smokers (n=24)	Effect (95% CI)	Test	p	FDR p
Gestational age (weeks)	38.20 ± 3.14	36.40 ± 4.74	-1.81 (-4.00, +0.39)	Welch t-test	0.104	0.208
Birth weight (g)	3141.67 ± 743.59	2827.92 ± 944.26	-313.75 (-767.31, +139.81)	Welch t-test	0.17	0.255
Apgar, 1 min (score)	8.71 ± 1.88	8.71 ± 1.43	-0.01 (-0.83, +0.82)	Welch t-test	0.988	0.988
Apgar, 5 min (score)	9.19 ± 1.63	9.25 ± 0.94	+0.06 (-0.57, +0.69)	Welch t-test	0.851	0.988
Preterm birth, n (%)	7 (16.7%)	9 (37.5%)	unadjusted OR 3.00	Fisher exact	0.076	0.208
Low birth weight, n (%)	5 (11.9%)	8 (33.3%)	unadjusted OR 3.70	Fisher exact	0.053	0.208

Continuous and categorical birth outcomes were compared between non-smokers and smokers using Welch's t-test and Fisher's exact test, respectively, with FDR-adjusted p-values reported across outcomes.

Abbreviations: CI, confidence interval; OR, odds ratio; FDR, false discovery rate. Continuous variables are presented as mean +/- standard deviation, categorical variables as number and percentage, continuous-outcome effects as smoker-minus-non-smoker mean differences, and binary-outcome effects as unadjusted odds ratios.

Table S11. Exploratory associations between composite redox indices and birth outcomes stratified by maternal smoking status.

Stratum	Index	Outcome	Events / n	Estimate (95% CI)	p	Reportable	FDR p
Non-smokers (n=42)	MOSI	Birth weight (g)	n=42	+22.8 (-228.0, +273.7)	0.855	yes	0.855
Non-smokers (n=42)	MOSI	Preterm birth	7/42	OR 0.40 (0.16, 1.03)	0.057	yes (exploratory)	0.231
Non-smokers (n=42)	MOSI	Low birth weight	5/42	OR 0.83 (0.32, 2.16)	0.699	yes (exploratory)	0.741
Non-smokers (n=42)	MNSI	Birth weight (g)	n=42	+90.2 (-44.0, +224.5)	0.182	yes	0.328
Non-smokers (n=42)	MNSI	Preterm birth	7/42	OR 0.36 (0.16, 0.85)	0.019	yes (exploratory)	0.172
Non-smokers (n=42)	MNSI	Low birth weight	5/42	OR 0.39 (0.15, 0.99)	0.048	yes (exploratory)	0.231
Non-smokers (n=42)	MGRI	Birth weight (g)	n=42	+76.8 (-42.9, +196.4)	0.202	yes	0.331
Non-smokers (n=42)	MGRI	Preterm birth	7/42	OR 0.35 (0.17, 0.74)	0.006	yes (exploratory)	0.113
Non-smokers (n=42)	MGRI	Low birth weight	5/42	OR 0.56 (0.30, 1.03)	0.064	yes (exploratory)	0.231
Non-smokers (n=42)	NOSI	Birth weight (g)	n=42	+104.4 (-34.4, +243.2)	0.136	yes	0.314
Non-smokers (n=42)	NOSI	Preterm birth	7/42	OR 0.85 (0.54, 1.32)	0.460	yes (exploratory)	0.533
Non-smokers (n=42)	NOSI	Low birth weight	5/42	OR 0.71 (0.44, 1.14)	0.158	yes (exploratory)	0.317
Non-smokers (n=42)	NNSI	Birth weight (g)	n=42	+57.3 (-81.5, +196.1)	0.409	yes	0.533
Non-smokers (n=42)	NNSI	Preterm birth	7/42	OR 0.80 (0.46, 1.38)	0.414	yes (exploratory)	0.533
Non-smokers (n=42)	NNSI	Low birth weight	5/42	OR 0.79 (0.42, 1.49)	0.474	yes (exploratory)	0.533
Non-smokers (n=42)	NGRI	Birth weight (g)	n=42	+73.4 (-19.2, +165.9)	0.117	yes	0.314
Non-smokers (n=42)	NGRI	Preterm birth	7/42	OR 0.83 (0.58, 1.18)	0.293	yes (exploratory)	0.44
Non-smokers (n=42)	NGRI	Low birth weight	5/42	OR 0.72 (0.47, 1.11)	0.140	yes (exploratory)	0.314
Smokers (n=24)	MOSI	Birth weight (g)	n=24	+340.5 (+154.3, +526.7)	<0.001	yes	0.009
Smokers (n=24)	MOSI	Preterm birth	9/24	OR 0.45 (0.23, 0.89)	0.022	yes (exploratory)	0.05
Smokers (n=24)	MOSI	Low birth weight	8/24	OR 0.51 (0.27, 0.95)	0.033	yes (exploratory)	0.06
Smokers (n=24)	MNSI	Birth weight (g)	n=24	+308.3 (-4.0, +620.7)	0.053	yes	0.079

Stratum	Index	Outcome	Events / n	Estimate (95% CI)	p	Reportable	FDR p
Smokers (n=24)	MNSI	Preterm birth	9/24	OR 0.36 (0.13, 0.98)	0.046	yes (exploratory)	0.075
Smokers (n=24)	MNSI	Low birth weight	8/24	OR 0.27 (0.08, 0.90)	0.033	yes (exploratory)	0.06
Smokers (n=24)	MGRI	Birth weight (g)	n=24	+271.5 (+140.5, +402.6)	<0.001	yes	0.005
Smokers (n=24)	MGRI	Preterm birth	9/24	OR 0.45 (0.24, 0.84)	0.012	yes (exploratory)	0.045
Smokers (n=24)	MGRI	Low birth weight	8/24	OR 0.46 (0.25, 0.85)	0.013	yes (exploratory)	0.045
Smokers (n=24)	NOSI	Birth weight (g)	n=24	-31.6 (-315.4, +252.2)	0.819	yes	0.819
Smokers (n=24)	NOSI	Preterm birth	9/24	OR 0.91 (0.51, 1.62)	0.738	yes (exploratory)	0.781
Smokers (n=24)	NOSI	Low birth weight	8/24	OR 0.90 (0.50, 1.64)	0.736	yes (exploratory)	0.781
Smokers (n=24)	NNSI	Birth weight (g)	n=24	+399.0 (+108.5, +689.5)	0.009	yes	0.045
Smokers (n=24)	NNSI	Preterm birth	9/24	OR 0.20 (0.05, 0.75)	0.017	yes (exploratory)	0.05
Smokers (n=24)	NNSI	Low birth weight	8/24	OR 0.21 (0.05, 0.78)	0.020	yes (exploratory)	0.05
Smokers (n=24)	NGRI	Birth weight (g)	n=24	+116.6 (-71.6, +304.7)	0.212	yes	0.255
Smokers (n=24)	NGRI	Preterm birth	9/24	OR 0.61 (0.35, 1.06)	0.078	yes (exploratory)	0.108
Smokers (n=24)	NGRI	Low birth weight	8/24	OR 0.60 (0.33, 1.08)	0.086	yes (exploratory)	0.111

Within-stratum regression estimates and FDR-adjusted p-values are reported separately for non-smokers and smokers, with binary-outcome models interpreted cautiously because of the small numbers of preterm and low-birth-weight events.

Abbreviations: OR, odds ratio; CI, confidence interval; FDR, false discovery rate; MOSI, maternal oxidative stress index; MNSI, maternal nitrosative stress index; MGRI, maternal glutathione redox index; NOSI, neonatal oxidative stress index; NNSI, neonatal nitrosative stress index; NGRI, neonatal glutathione redox index. Birth-weight estimates are regression coefficients in grams, whereas preterm-birth and low-birth-weight estimates are odds ratios; 'reportable' indicates that an estimate was obtained but does not establish model reliability or confirmatory significance.

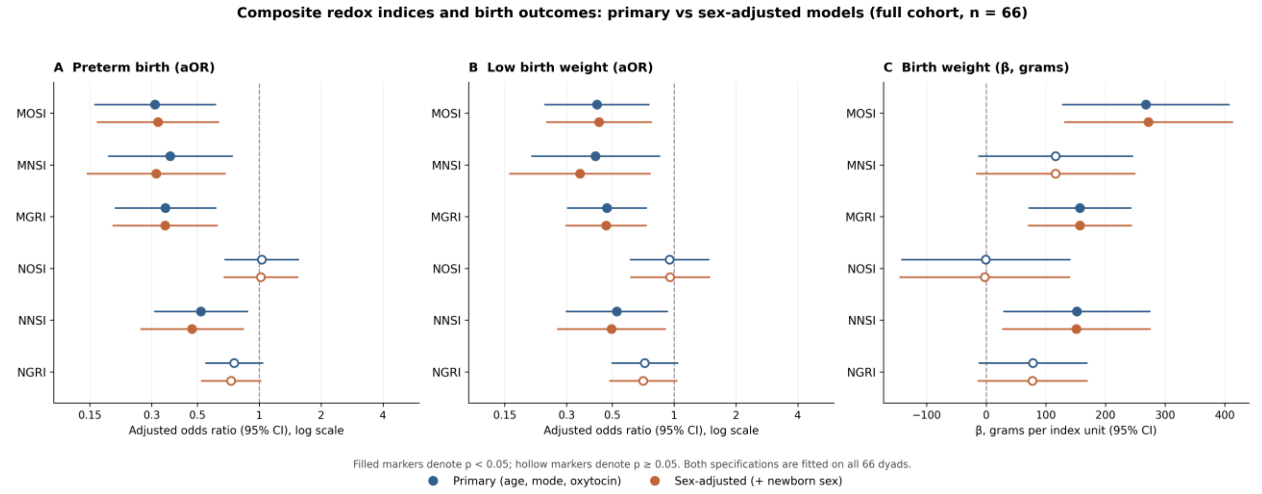

Figure S1. Primary and newborn-sex-adjusted associations between composite redox indices and birth outcomes. Forest plots compare primary and newborn-sex-adjusted estimates and 95% confidence intervals for associations of maternal and neonatal composite redox indices with birth weight, preterm birth, and low birth weight. Points represent model estimates and horizontal lines represent 95% confidence intervals; birth-weight associations are expressed as regression coefficients in grams, whereas preterm-birth and low-birth-weight associations are expressed as adjusted odds ratios. Abbreviations: MOSI, maternal oxidative stress index; MNSI, maternal nitrosative stress index; MGRI, maternal glutathione redox index; NOSI, neonatal oxidative stress index; NNSI, neonatal nitrosative stress index; NGRI, neonatal glutathione redox index.

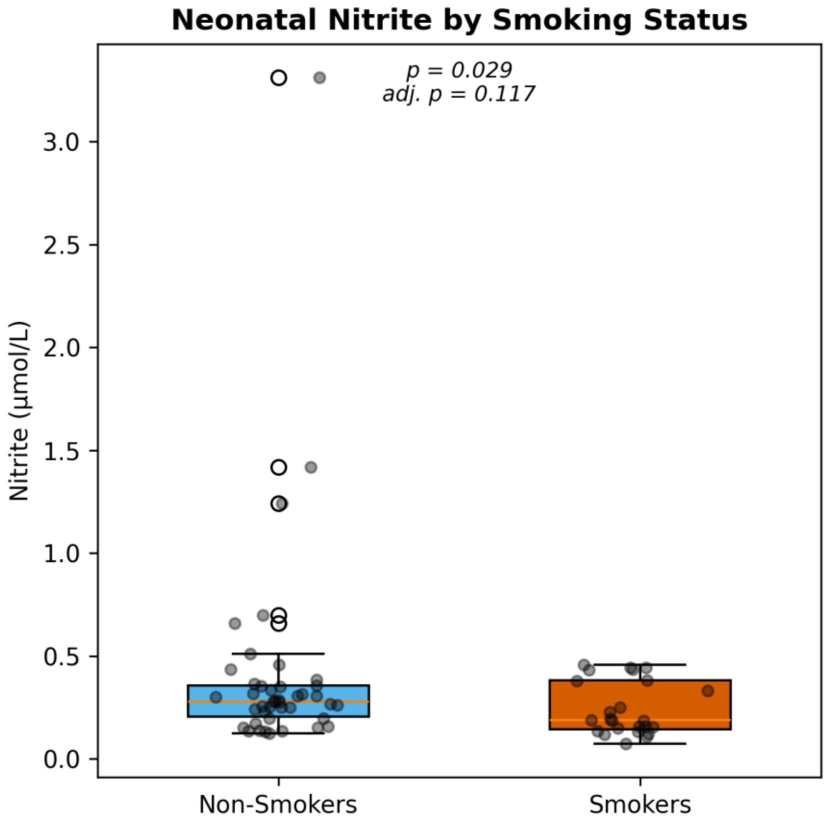

Figure S2. Neonatal nitrite concentrations according to maternal smoking status. The distribution of neonatal nitrite concentrations is compared between newborns of non-smokers and smokers in an exploratory component-level analysis. Graphical elements should be interpreted as displayed in the figure; the corresponding unadjusted group comparison is reported in Table S5.

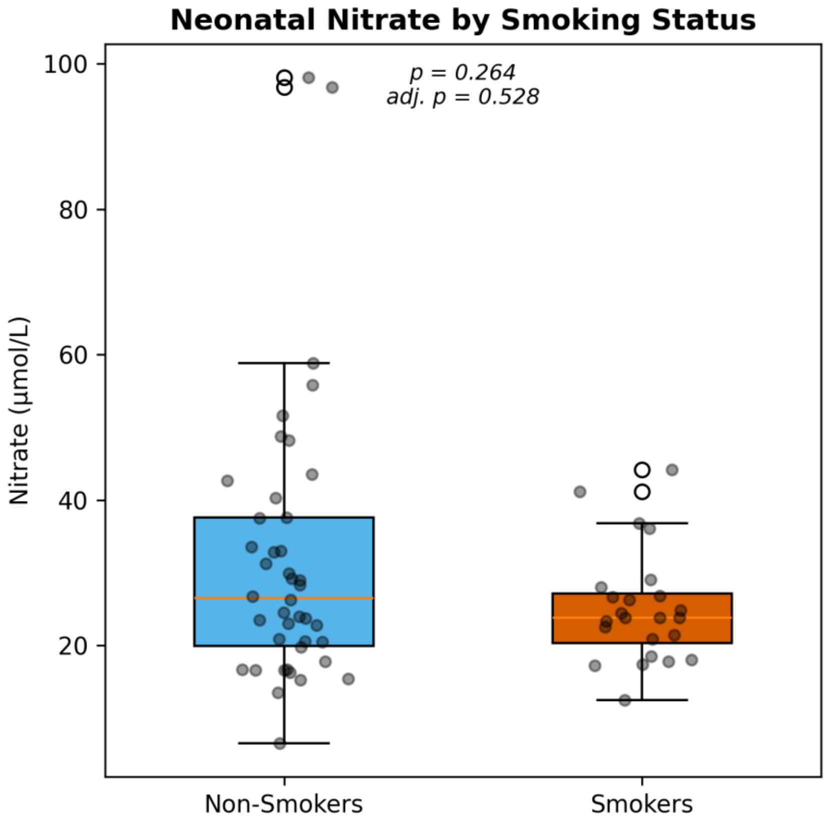

Figure S3. Neonatal nitrate concentrations according to maternal smoking status. The distribution of neonatal nitrate concentrations is compared between newborns of non-smokers and smokers in an exploratory component-level analysis. Graphical elements should be interpreted as displayed in the figure; the corresponding unadjusted group comparison is reported in Table S5.

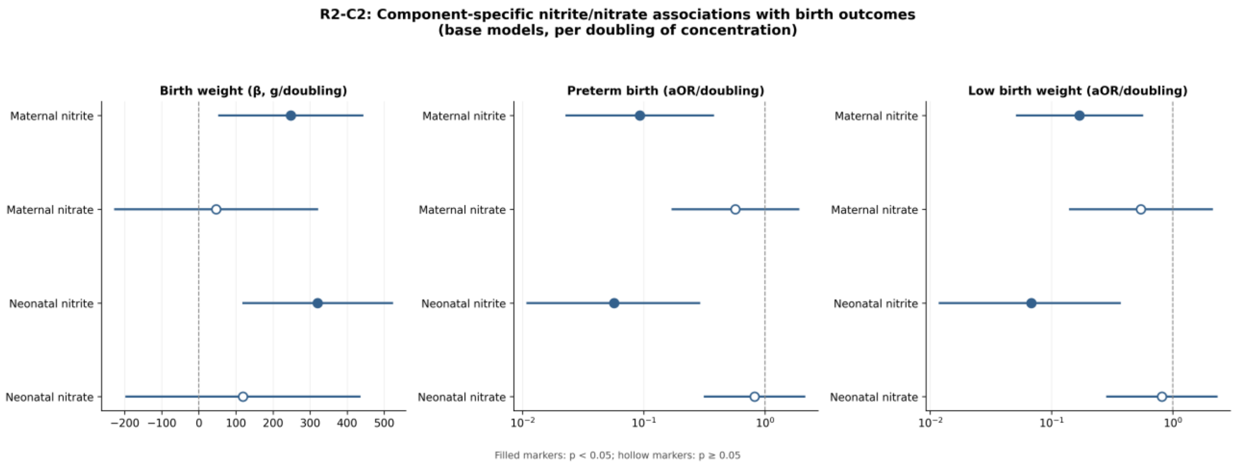

Figure S4. Component-specific associations of maternal and neonatal nitrite and nitrate with birth outcomes. Forest plots summarize exploratory associations of maternal and neonatal nitrite and nitrate with birth weight, preterm birth, and low birth weight in base and maternal-smoking-adjusted models. Points represent

model estimates and horizontal lines represent 95% confidence intervals; birth-weight effects are expressed as regression coefficients in grams per doubling of biomarker concentration, and binary-outcome effects as adjusted odds ratios per doubling. Abbreviations: PTB, preterm birth; LBW, low birth weight; CI, confidence interval; estimates are exploratory because of the small sample and low event counts.
